# Supplementary material for: Influence of different sample preparation approaches on proteoform identification by top-down proteomics
Source: Nat Methods. 2024 Oct 22;21(12):2397–407. doi: 10.1038/s41592-024-02481-6 (PMC11621018; doi:10.1038/s41592-024-02481-6)
Supplement: Supplementary file 1 — Supplementary Results, Notes, Figs. 1–26, Tables 1–3, 7 and 8 and References. [file 41592_2024_2481_MOESM1_ESM.pdf]

nature methods

Supplementary information https://doi.org/10.1038/s41592-024-02481-6

Influence of different sample preparation approaches on proteoform identification by top-down proteomics

In the format provided by the authors and unedited

Table of Contents

Table of Contents **1**

Supplementary Results **2**

Optimization LC-MS/MS Workflow 2
FAIMS-MS/MS Settings 2
Repeatability 3
Influence of the Injection Amount 5
Influence of the Enrichment of Suitable Proteoforms 6
Quality Control 7
Modified Proteoforms 7
Identified Proteins 8
Variations of the Proteoform Isolation Strategies 8
Proteoform Fractionation 10
Fractionation Efficiency 10
Proteoforms Identified in this Study 11

Supplementary Notes: Guidelines for the Sample Preparation in TDP **13**

Supplementary Notes: Various Observations **16**

Supplementary Figures **18**

Supplementary Tables **45**

Supplementary References **53**

Supplementary Results

Optimization LC-MS/MS Workflow

FAIMS-MS/MS Settings

In order to cover a broad proteoform mass range, two different LC-FAIMS-MS workflows utilizing internal compensation voltage (CV) stepping were used.[1] For targeting proteoforms smaller than ca. 15 kDa (from now on referred to as the LMW method), the CVs −60, −50, −40, and −25 V were selected. Moreover, the acquisition was performed in peptide mode, i.e., with a higher pressure in the ion routing multipole (IRM), and CID fragmentation was utilized. In contrast, to target proteoforms larger than ca. 15 kDa (HMW method), the CVs −30, −20, 0, and +15 V were selected, and fragmentation was performed with EThcD. Moreover, the acquisition was performed in protein mode (lower pressure in the IRM), and a medium/high acquisition strategy was applied.[2]

Ten technical replicates of a proteome derived from a cell lysate of human Caco-2 cells (enriched with proteoforms smaller than 20 kDa by SPE using C18 material)[3] were analyzed to examine the two methods in detail. The various CVs of the LMW and HMW methods resulted in different total ion chromatogram (TIC) profiles, highlighting the effectiveness of gas-phase separation by FAIMS (**Supplementary Figure 2A**). Notably, the combination of FAIMS with EThcD fragmentation resulted in a jagged TIC, i.e., the intensity of the consecutive MS1 signals varied considerably. According to the instrument vendor, Thermo Fisher Scientific, the reason for this is that the FAIMS DC offset is shared with the stacked ring ion guide (SRIG) DC. However, the SRIG DC offset is not restored to the precursor polarity on time to stabilize the precursor signal after an ETD reagent injection. Upon manual inspection, it was found that this did not affect the overall quality of the acquired MS2 spectra, but it may have an impact on label-free quantification. For proteoform identification, the raw files were analyzed by ProSightPD (FDR<1%). Only proteoforms with a C-score above 40 were considered to obtain well-characterized proteoform identifications.[4]

The LMW and HMW methods identified a similar number of proteoforms (1,515 and 1,384, respectively), but more proteins were identified by the LMW (515) compared with the HMW method (215) (**Supplementary Figure 3**). The LMW method identified more truncated

(subsequence) proteoforms than the HMW method; nevertheless, the majority of the proteoform spectral matches (PrSMs) were assigned to annotated (full-length) proteoforms in both methods. We adopted the definition of annotated proteoforms from ProSightPD, i.e., annotated proteoforms are all full-length proteoforms deposited in the database, including, e.g., start-methionine and signal peptide excision. In contrast, truncated proteoforms are proteoforms that have been truncated in a way that is not described in the database.

Using the HMW method, the proteoform mass ranged from 5.7-57.9 kDa, with a median of 15.4 kDa (**Supplementary Figure 1A**). Compared to that, the LMW method identified much smaller proteoforms in the mass range of 2.1-26.6 kDa, with a median of 6.1 kDa. Consistent with the literature,[2] the low-resolution MS1 spectra enabled the detection of large proteoforms possessing charge states up to 57^+ (**Supplementary Figure 2B-D**); however, the isotopic unresolved spectra led to lower accuracy in precursor mass determination compared to high-resolution data. Thus, the maximum precursor tolerance for identifying annotated proteoforms in the medium/high datasets was set to 2.2 Da.[2,5] In contrast, the LMW method enabled high mass accuracy and the resolution of the isotopic distribution, which are valuable tools for manual proteoform validation (**Supplementary Figure 2E/F**).[6]

The high complementarity of the two methods was demonstrated by the low overlap regarding identified proteoforms (10%) and proteins (25%) (**Supplementary Figure 1A**, **Supplementary Figure 3C**). The LMW method identified slightly more acidic and hydrophilic proteoforms than the HMW method (**Supplementary Figure 3D**). Furthermore, consistent with previous studies,[7] proteoforms identified with both methods showed a higher residue cleavage after EThcD fragmentation utilized in the HMW method compared to the CID fragmentation used in the LMW method (**Supplementary Figure 4**).

In summary, the two MS methods provide complementary proteoform identifications attributed to the different FAIMS settings, acquisition strategies, and fragmentation mechanisms, enabling the characterization of proteoforms within a wide mass range.

Repeatability

Due to instrument variance, i.e., slight variations of retention times and MS performance, multiple injections of the same sample can result in different identifications.[8] For a fair comparison between several sample preparation steps, it is essential to investigate the repeatability of technical replicates to distinguish between differences originating from

instrument variance and the actual sample preparation.[9] In addition, evaluating the variability of LC-MS/MS allows a rational decision on how many replicates are required for each sample, ensuring a high number of identifications while also avoiding excessive measurement time. To this end, the datasets acquired to examine the HMW and LMW methods were evaluated regarding their reproducibility.

On average, the LMW method identified 621±15 proteoforms from 320±8 proteins (average ± standard derivation, n=10) (**Supplementary Figure 5A/B**), with an overlap coefficient between the replicates of approximately 79% on the proteoform and 88% on the protein level (**Supplementary Figure 1B**), demonstrating a high reproducibility. Interestingly, the overlap coefficients of the annotated proteoforms (89%) were significantly higher than those from the truncated proteoforms (75%), which could possibly be explained by a higher abundance of annotated proteoforms (**Supplementary Figure 5C**). The number of assigned PrSMs correlates with the proteoform abundance and, thus, can be used as a rough semi-quantitative approach.[10] Overall, 79±1% of the PrSMs were assigned to annotated, and only 21±1% were assigned to truncated proteoforms. The higher abundance of annotated proteoforms translates to an increased likelihood of selection for fragmentation and subsequently leads to more reproducible identification. Notably, the quality and the physicochemical properties of the identified proteoforms, such as isoelectric point, proteoform masses, and GRAVY score, were very similar for all replicates (**Supplementary Figure 5D-G)**.

Next, we examined the gain of multiple injections regarding the number of identifications (**Supplementary Figure 1C, Supplementary Figure 5H**).[9,11] On average, the second injection increased the proteoform identifications by 21% and the protein identifications by 13% compared to a single injection. The third injection added, on average, 10% proteoforms and 7% proteins. Additional measurements led to a successive decreasing increment of identifications; for example, only 3% of additional proteoforms and 2% of proteins were identified after seven cumulative injections.

The reproducibility of the HMW method was slightly worse than that of the LMW method (**Supplementary Figure 6**). For example, the overlap coefficient between two replicates was between 58-69%, mainly attributed to the lower reproducibility of truncated proteoforms (overlap coefficient of approximately 37%, compared to 79% for annotated proteoforms). This observation can possibly be explained by the bias against detecting small proteoforms with a

low-resolution MS1 acquisition method. Low-resolution MS1 data require deconvolution via the detection of charge patterns, which is more accurate when more charge states of a proteoform are present. Small proteoforms, however, typically have a reduced number of detectable charge states compared to larger proteoforms. With the HMW method, the second injection increased, on average, the proteoform and protein identifications by 35% and 16%, whereas the third injection added 20% and 8%, respectively. The reproducibility of the HMW method regarding the number of identifications, physicochemical properties, and confidence of the identifications was very high, similar to the LMW method.

Based on the technical repeatability analysis, we performed three replicates per sample for the following investigations utilizing the LMW and HMW methods to increase the number of proteoform identifications while keeping the measurement time moderate. Notably, the number, confidence, and physicochemical properties of the identified proteoforms were highly reproducible within technical replicates (**Supplementary Figure 5, 6**).

Influence of the Injection Amount

Different sample preparations yield different protein quantities that can be injected for LC-MS/MS analysis, which can affect the depth of the analysis.[12] On the one hand, too low injection amounts may prevent the identification of low abundant proteoforms. On the other hand, too high injection amounts may result in non-optimal chromatographic separation caused by broad and overlapping peaks, as well as by column overloading. Thus, the influence of the injection amount on the number and confidence of proteoform identifications was investigated. For this, 30-1,200 ng of a proteome derived from human Caco-2 cell lysate (enriched with small proteoforms by SPE) were analyzed in triplicates by LC-MS/MS using the LMW method.

In general, the higher the injection amount, the higher the number of identifications, ranging from 197±10 to 734±8 proteoforms and 137±8 to 360±15 proteins (average ± standard derivation, n=3) at 30 ng and 1,200 ng, respectively (**Supplementary Figure 1D**). The overlap coefficient between all injections was in the same range as for technical replicates, demonstrating that with decreasing sample amounts, only a subset of abundant proteoforms was identified (**Supplementary Figure 7A/B**).

Besides the number of identifications, the confidence of proteoform identifications was also improved with increasing injection amount. For example, the average residue cleavage

increased from 19±1% (30 ng injection) to 30±1% (900 ng injection) (**Supplementary Figure 7C**). In addition, the higher the sample amount, the higher the number of modified proteoforms identified; e.g., an injection of 30 ng resulted in only 14 serine-phosphorylated proteoforms, whereas more than five times more (72) were identified with an injection of 1,200 ng (**Supplementary Figure 7D**).

Interestingly, the ratio of the annotated compared to truncated proteoforms decreased with increasing sample injection amount (**Supplementary Figure 7E/F**). With a 30 ng injection, approximately the same number of annotated (101±9) and truncated proteoforms (96±4) were identified. In contrast, the injection of 1.2 µg results in twice as many truncated (517±7) than annotated proteoforms (217±3). The same trend, even with a higher degree, was observed for the number of assigned PrSMs. This observation could possibly be explained by the lower abundance of truncated proteoforms, which is consistent with the repeatability studies of LC-MS/MS measurements described above.

Based on these results, it is evident that injecting approximately the same amount of proteoforms for LC-MS/MS analyses is essential to ensure a fair comparison of different sample preparation methods. Therefore, in this study, a similar injection volume was injected for all experiments based on the total number of ions.

Influence of the Enrichment of Suitable Proteoforms

Human Caco-2 cells were lysed in PBS buffer, with the same batch of Caco-2 cells used for all experiments. Aliquots were purified by MCW precipitation and subjected to various sample preparation strategies using the established LC-MS/MS workflow. All experiments were performed in triplicates to increase the number of identifications and examine the reproducibility. For the isolation of proteoforms by MWCO filters, the sample was applied to a 30 kDa filter and centrifuged.[13] Thus, theoretically, only proteoforms below the MWCO size can pass the filter. Furthermore, size-based fractionation strategies were performed to enrich proteoforms smaller than approximately 30 kDa. In the PEPPI protocol,[14] proteoforms are separated by SDS-PAGE and eluted from the gel through passive elution. In contrast, the GELFrEE system recovers the proteins in solution after gel electrophoresis.[15] Another size-based separation method is SEC, which was conducted with an acidic organic-aqueous mobile phase. Besides that, we applied an acidic acetonitrile-based depletion strategy,[16–18] specifically depleting proteoforms larger than 15-30 kDa, whereas small proteoforms remain in solution.

For SPE sample preparation, the proteome was loaded on a C18 SPE cartridge, and the bounded proteoforms were washed with an acidic water solution.[3] Subsequently, proteoforms were eluted with a high content of organic solvents.

Quality Control

The injection amount of all samples was adjusted to achieve a TIC of ca. $3\text{-}6\times10^9$. Strikingly, the profiles and most intense signals in the TICs were similar in all raw files of the different approaches, with the exception of the acetonitrile depletion, which showed a different TIC profile (**Supplementary Figure 12A**). The retention time stability across the entire measurement series was reasonable based on randomly selected precursors at the start (variation approximately smaller than 1.5 min), middle (variation <2 min), and end (variation <3 min) of the gradient (**Supplementary Figure 12B-D**).

Further, we examined the confidence of the proteoform identified by the various sample preparation strategies, including the C-score, *E*-value, *p*-score, and residue cleavage (**Supplementary Figure 13**). Only slight differences were observed; for example, the highest average C-score was obtained with the GELFrEE approach (1,241), whereas the highest residue cleavage was obtained for proteoforms identified after acetonitrile depletion (29.8%).

Modified Proteoforms

Generally, the number of modified proteoforms correlated with the total number of identifications, i.e., the more identified proteoforms, the more modifications were detected. For example, proteoform isolation by MWCO filters resulted in the identification of 178 phosphorylations on serine residues and 19 N-terminal myristoylations. After the gel-based approaches, the number of identified proteoforms with oxidized cysteine residues was low (GELFrEE: 18, PEPPI: 41), which might be explained by the reduction of the proteoforms using β-mercaptoethanol or dithiothreitol prior to electrophoresis. The fact that disulfides were still identified is in agreement with the results of the investigations of reduction/alkylation of the proteoforms and can be explained either by incomplete reduction or re-oxidation of the cysteine residues during sample preparation.

Besides biological modifications, we investigated the occurrence of artificial modifications introduced during sample preparation and downstream analytics. To this end, the raw files were

deconvolved with FLASHDeconv, and the mass features were analyzed with MSTopDiff (**Figure 5D**). In all approaches, mass shifts of 16 Da, 32 Da, and 48 Da that could be assigned to (multiple) oxidation events were observed. In the PEPPI dataset, adducts from β-mercaptoethanol (Δm=60.01 Da, 2-OH-ethyl thio-serine, and Δm=76.00 Da, cysteine mercaptoethanol) were observed, an artifact from incubating the sample in Laemmli buffer prior to SDS-PAGE separation. It should be noted that reduction/alkylation of the sample prior to separation with PEPPI, as envisaged in the original PEPPI protocol,[14] would prevent the formation of β-mercaptoethanol adducts. Furthermore, employing reductive agents such as β-mercaptoethanol is not mandatory and can be replaced with other alternatives such as DTT or completely omitted. The SPE sample preparation resulted in the identification of a formylation-related mass shift (Δm=28.00 Da), which can be explained by the use of formic acid at ambient temperature.

Identified Proteins

Among the ten most abundant protein sequences identified (based on PrSM count), there was considerable overlap between the investigated approaches, except for acetonitrile depletion. Notably, the acetonitrile depletion resulted in a bias towards histone proteins; e.g., six of the ten most abundant proteins belong to histone proteins. The tubulin-specific chaperone (UniProt accession: O75347-1) was identified among the most abundant proteins in all samples. Besides that, the mitochondrial 10 kDa heat shock protein (P61604), ribosomal proteins (P05387, P39019), parathymosin (P20962), S-100 proteins (P31949, P60903), Calmodulin (P0DP23), and triosephosphate isomerase (P60174-1) were identified with a high abundance in all approaches, except after acetonitrile depletion. Interestingly, the Histone H4 protein (P62805) was the most abundant protein in all datasets but the MWCO dataset, where it was not even in the top ten.

Variations of the Proteoform Isolation Strategies

Several variations of the investigated sample preparation strategies were examined (**Supplementary Figure 17**). The SPE protocol was initially performed with reversed-phase C18 phases[3] and further studied using C4 phases. Compared to the C18, the C4 protocol

resulted in almost identical identifications regarding the number, confidence, reproducibility, and physicochemical properties (proteoform mass, GRAVY score, and *pI*).

The acetonitrile depletion method can be performed either under acidic (ACN/NaCl with 0.1% TFA) or basic (ACN/TEAB, pH 8.5) conditions. Compared to the acidic depletion described above, the basic depletion resulted in a significantly lower number of identifications, with 401±46 proteoforms and 225±24 proteins (n=3). Strikingly, significantly more proteoforms with an acidic *pI* were identified using the ACN/TEAB approach, resulting in a median *pI* of 7.2 (ACN/NaCl: 9.8). This observation perfectly aligns with the results described above about the influence of the pH on the *pI* of the proteoforms identified. Furthermore, the identified proteoforms were more hydrophobic using the ACN/TEAB method (GRAVY score of −0.27) compared to the ACN/NaCl protocol (−0.54).

Besides 30 kDa MWCO filters, 50 kDa and 100 kDa filters are often utilized. However, applying the MWCO protocol described in the Material and Method section to 100 kDa filters, the subsequent LC-MS/MS analysis resulted in a critical column pressure profile, i.e., the pressure increased successively and showed jumps up to 50 bar. Finally, the analysis of the samples prepared with 100 kDa filters clogged the ESI emitter; therefore, these filters were not further investigated. The 50 kDa filter led to fewer identifications (546±21 proteoforms from 284±14 proteins) compared to the 30 kDa MWCO filter. Furthermore, the identified proteoforms were more hydrophilic, with a median GRAVY score of −0.7 (30 kDa MWCO filter: −0.56).

An optimized protocol for analyzing small proteoforms has recently been developed for the PEPPI fractionation, utilizing anion-exchange stage-tip (anion-exchange disc-assisted sequential sample preparation, AnExSP) purification instead of MCW precipitation].[19,20] In agreement with the literature, AnExSP identified more small proteoforms due to avoiding the precipitation step.[20,21] However, fewer proteoforms larger than 10 kDa were identified, resulting in an overall reduced number of identifications (543±123 proteoforms from 289±58 proteins). The average proteoform size using PEPPI-AnExSP was significantly reduced (6.6 kDa) compared to PEPPI-MCW (8.2 kDa). Similar to PEPPI-MCW, PEPPI-AnExSP showed a bias towards more acidic proteoforms.

Proteoform Fractionation

A recently developed two-dimensional low/low pH reversed-phase LC separation scheme was utilized to fractionate the proteome of Caco-2 cells in the first dimension into 48 fractions, which were subsequently concatenated into eight pools.[22] As a gel-based method, GELFrEE was employed, fractionating eight fractions below approximately 50 kDa. All experiments were performed in triplicates, and the samples were analyzed with two injections using the LMW and HMW methods, respectively.

It is worth noting that another common and highly orthogonal method for separating intact proteoforms is the high/low pH strategy, which involves first fractionating the proteoforms with high-pH reversed-phase LC, followed by separation with low-pH reversed-phase LC before MS analysis.[23,24] Recently, we compared the low/low pH and the high/low pH separation schemes and demonstrated that the low/low pH separation scheme outperformed the high/low pH separation in terms of the number of identifications.[22] However, the high/low and low/low pH separation schemes resulted in highly complementary proteoform identifications.

Fractionation Efficiency

The fractionation efficiency and the properties of the different fractions were examined in more detail for both approaches. In the LC-based approach, all pools were quite similar regarding their number of identifications, ranging from 325±32 to 489±71 proteoforms (fractions 4 and 6, respectively) and 186±8 to 225±28 proteins (fractions 8 and 6, respectively) (**Supplementary Figure 20A**). The overlap between two adjacent pools in the LC-based fractionation schemes was approximately 30% on proteoform and 50% on protein level (**Supplementary Figure 20B**). In addition, 73% of the proteoforms were uniquely identified in only a single fraction, demonstrating the high separation efficiency (**Supplementary Figure 20C**). No differences between the various fractions regarding the physicochemical properties (proteoform *pI*, mass, or isoelectric point) were observed. This is in accordance with previous reports and can attributed to the applied concatenation strategy (**Supplementary Figure 20D-F**).[22]

The gel-based fractionation showed a slightly worse separation efficiency compared to the LC-based fractionation (**Supplementary Figure 21A-C**). The number of identifications was more diverse, ranging from 80±9 to 606±314 proteoforms (fractions 1 and 6, respectively) and 41±10 to 272±35 proteins (fractions 1 and 4, respectively). In particular, the first three fractions had

a relatively high proteoform overlap (60-75%), while the overlap of adjacent fractions was approximately 40%. About 69% of the proteoforms were identified in only a single fraction, which is in a similar range as in the LC-based fractionation. Due to the size-dependent fractionation, the median proteoform size increased with increasing fraction number (**Supplementary Figure 21D**), from 7.9 kDa in fraction 1 to 20 kDa in fraction 6. Although most large proteoforms were identified in fractions 7 and 8, many small proteoforms were also identified, so the average proteoform size was around 10 kDa. The observation of small proteoforms in fractions corresponding to higher mass ranges is in agreement with previous studies. The *pI* decreased with increasing fraction number, with more acidic proteoforms identified in later fractions. Compared to that, no clear trend was observed regarding the GRAVY score (**Supplementary Figure 21E/F**).

Proteoforms Identified in this Study

The most abundant proteoforms (based on PrSM count) were full-length proteoforms from Histone H4 (P62805), Calmodulin (P0DP23), and the Tubulin-specific Chaperone A (O75347). More than 1,380 protein sequences were identified with at least two different proteoforms; from 32 protein sequences, more than 100 proteoforms were identified (**Supplementary Figure 22A**). Note that we here cannot distinguish between biologically formed proteoforms and such formed due to artifacts during analysis (e.g., artificial truncations).

Due to the high sequence coverage, several proteoforms carrying modifications on different residues could be unambiguously identified. For example, the guanine nucleotide-binding protein (P63218) was identified with six proteoforms, including a S-geranylgeranyl cysteine, a C-terminal truncated form (removal of the propeptide) in combination with geranylgeranyl cysteine and cysteine methyl ester, respectively, and four previously undescribed C-terminal truncations (**Supplementary Figure 24**).

TDP inherently provides information about the N- and C-terminus of the proteoforms. Analyzing the identified canonical N-termini revealed that the start methionine was typically excised when the adjacent amino acid was glycine, alanine, proline, serine, threonine, cysteine, or valine (**Supplementary Figure 26A/B**). In contrast, the start-methionine was typically not excised if the amino acid in the second position was aspartate, glutamate, lysine, arginine, leucine, isoleucine, phenylalanine, asparagine, tryptophan, or glutamine. This observation is in

agreement with previous studies, reporting that the start-methionine is processed by the methionine aminopeptidase based on the size of the adjacent residue.[25] Approximately 30% of the proteoforms were N-terminally (excluding start methionine excision), 32% C-terminally, 24% N- and C-terminally truncated, and 14% were annotated full-length proteoforms. The truncated proteoforms showed diverse potential cleavage sites (**Supplementary Figure 26C/D**). Several proteoforms with truncated signal peptides were identified, such as mitochondrial proteins from the Cytochrome c oxidase (P15954, P14406, P12074, P24311, P20674, P10606, P10176, P13073,) confirming the annotated signal peptides. In some cases, the deposited signal peptide in UniProt[26] differed from the identified proteoform by a few amino acids. For example, the mitochondrial ATPase inhibitor (Q9UII2) was identified with a cleaved signal peptide deposited in UniProt and several proteoforms that are one or a few amino acids longer and shorter, respectively.

Supplementary Notes: Guidelines for the Sample Preparation in TDP

The decision tree visualized in **Figure 6** (main manuscript) provides specific recommendations for the selection of a workflow based on the objective of a qualitative TDP study. Two possible examples to explain the use of the decision tree: (i) The targets of a study are small open reading frame-encoded peptides or antimicrobial peptides, i.e., subgroups of proteoforms smaller than 10 kDa, which typically have alkaline *pI*.[27,28] In this case, the decision tree guides to a sample preparation strategy utilizing acidic organic depletion. Furthermore, following the general recommendations described in the main manuscript and this chapter (below), cell lysis should be performed using an acidic pH. (ii) The study aims to map the proteoforms of a sample with several milligrams of available total protein. Here, two-dimensional separation schemes and complementary methods can be recommended, such as filter-based, organic depletion, PEPPI, and SPE-based sample preparation. The choice of the number and combination of parallel approaches performed can be supported by **Figure 4E/F** (main manuscript).

The general recommendations are valid for all steps of sample preparation, i.e., cell lysis, sample cleanup, reduction, alkylation, and the enrichment of suitable proteoforms. In the following, some critical factors of the various sample preparation steps and possible solutions are presented (summarized in **Supplementary Table 8**).

During **cell lysis**, critical factors are mild acidic conditions and heat, which can result in artificially truncated proteoforms due to hydrolysis of peptide bonds C-terminal to aspartate residues. For example, using a sonication probe for cell lysis can lead to heating and mechanical stress of the sample, potentially resulting in artificially truncated proteoforms or unwanted protein precipitation.[29] Possible solutions for these critical steps are using less intense lysis conditions (e.g., using an ultrasonic bath instead of a sonication probe; cooling is always recommended) and alkaline buffer systems. Moreover, protease inhibitors can introduce artificial modifications (e.g., covalent AEBSF adducts) and contamination peaks if certain compounds are enriched during the subsequent sample preparation. In this case, alternative protease inhibitors, lysis solutions, or sample preparations can be used.

The **sample cleanup** typically involves protein precipitation and re-solubilization. Several studies have demonstrated the influence of the sample cleanup strategy on the proteoform identifications.[20,21,30] For example, classical methanol-chloroform-water precipitation and

acetone precipitation have been shown to result in a significant loss of small proteoforms. To avoid this, optimized precipitation protocols (e.g., acetone precipitation with zinc sulfate),[31] or specialized cleanup strategies (e.g., proteoform purification using anion exchange)[20] can be utilized. To facilitate the protein resolubilization after precipitation, the protein pellet can be incubated in cold (−20 °C) concentrated (80%) formic acid and diluted tenfold with water prior to MS analysis.[32] Note that cooling of the sample is necessary to prevent artificially introduced formylation.[33]

Reduction of the proteoforms is often performed under elevated temperatures, which can lead to artificially truncated proteoforms due to hydrolysis C-terminal to aspartate residues. Therefore, heating should be avoided, e.g., by performing proteoform reduction with TCEP at room temperature.[34] Moreover, after reduction, proteoforms containing disulfides may still be identified, e.g., due to refolding of the disulfides during the sample preparation. A possible solution is to perform the reduction in combination with the subsequent alkylation of the cysteine residues.

In the gel-based approaches, **PEPPI and GELFrEE**, artificial modifications can be introduced, such as covalent β-mercaptoethanol or acrylamide adducts. Possible solutions to avoid these issues are using other reduction reagents, such as dithiothreitol, or performing reduction in combination with subsequent alkylation of the proteoform prior to the gel-based separation.

A critical step in the **SPE protocol** is using formic acid due to the risk of artificially introduced formylations. Working at low temperatures can minimize artificial modifications.[33] Moreover, replacing formic acid with another ion-pairing reagent, such as TFA,[35] prevents artificial formylations. During the SPE, some hydrophobic compounds of the samples, e.g., pepstatin A from the protease inhibitor, may be enriched. In this case, an alternative protease inhibitor or sample preparation strategy can be used.

Critical steps in the **2D-low/low pH-LC** separation scheme are elevated temperatures and prolonged exposure under acidic conditions, which can result in artificially truncated proteoforms.[22] Possible solutions for this issue are (i) performing sample concentration at low temperatures using lyophilization (instead of vacuum centrifugation at elevated temperatures),[22] (ii) buffering the fractions to basic pH immediately after the fractionation,[22] and (iii) performing alternative 2D separation approaches, such as the high/low pH separation scheme.[23] Note that similar to the situation in BUP, the use of multidimensional separation

schemes may cause problems in label-free quantitative studies, e.g., label-free quantification (LFQ) or Proteoform Reaction Monitoring (PfRM).[36]

Supplementary Notes: Various Observations

In the course of this study, several observations were made, which partially led to the failure of experiments. The description of these experiments can provide important insights into potential issues to be taken into account for the design of top-down proteomics experiments. In addition, various general observations during sample preparation or LC-MS/MS analysis are described.

- The solubilization of precipitated proteoforms in LC-MS loading (3% acetonitrile, 0.1% TFA) buffer for the 1D-LC control results in a significant protein loss, evident in insoluble precipitates. Thus, we tested the use of 8 M guanidinium hydrochloride and subsequent dilution with LC-MS loading buffer to increase the protein recovery. Although the solubilization was successful, i.e., no precipitate was observed after centrifugation, the injection of the sample for LC-MS/MS analysis resulted in (reproducible) clogging of the electrospray emitter. Notably, the emitter clogging often occurred not directly when the sample was injected but several hours/injections later. Furthermore, it is noteworthy that the clogging of the column or the electrospray emitter was frequently observed when the sample was not sufficiently cleaned and still contained salts, for example.
- The use of ETD fragmentation in combination with FAIMS resulted in issues with the spray stability and, thus, a jagged total ion chromatogram. Although this does not affect the overall quality of the MS2 spectra, it might influence label-free quantification. Note that we used the Thermo Scientific™ Orbitrap™ Tribrid™ Series 3.4 instrument control application (v3.4.3072.18) for all measurements.
- The isolation of proteoforms suitable for TDP was also investigated using 100 kDa MWCO filters. However, after performing the described protocol and subsequent LC-MS/MS analysis, the column pressure of the nano pump increased successively and showed jumps up to 50 bar. After that, the column could only be cleaned by excessive washing with Magic Mix (25:25:25:25 MilliQ/acetonitrile/methanol/isopropanol plus 0.1% formic acid).
- The reduction of proteoforms in PBS buffer resulted in a significant loss of proteins due to protein precipitation. Therefore, the reduction/alkylation of the proteoforms was performed in a buffered (200 mM triethylammonium bicarbonate; pH 8.5) 8 M GndHCl solution.
- Analyzing the raw data with MSTopDiff sometimes reveals artifactual mass shifts caused by the mass difference of contaminations (i.e., not due to the mass difference of modified/non-modified proteoforms). A typical contamination is PEG, resulting in an

intense mass shift at 44.03 Da and multiples thereof. In addition, mass shifts at 14.01 Da and 28.01 Da are frequently observed due to unknown contamination (note that, for example, the 28.01 Da peak at a bin size of 0.01 Da does not fit dimethylation (Δm= 28.031 Da)). To simplify the MSTopDiff analysis, PEG contamination, for example, can be easily excluded using the retention time mass feature filter since PEG typically elutes late in the chromatogram.[37]

- The performance of the LC-MS/MS instrument was monitored by injecting an in-house complex proteoform standard (*Escherichia coli* proteome enriched in small proteoforms by solid-phase extraction[3] supplemented with a six-protein standard (Thermo Fisher Scientific, product number A33526). The intensity of the TIC and the number of identified proteoforms were valuable indications of the instrument's performance. The in-house complex proteoform standard was analyzed approximately every 1-2 days. When a performance drop was observed, the front end of the mass spectrometer was cleaned according to the manufacturer's recommendations. Independently of this, the FAIMS electrodes were cleaned regularly every 1-2 weeks.
- Calibration of the instrument was performed approximately every two weeks.

Supplementary Figures

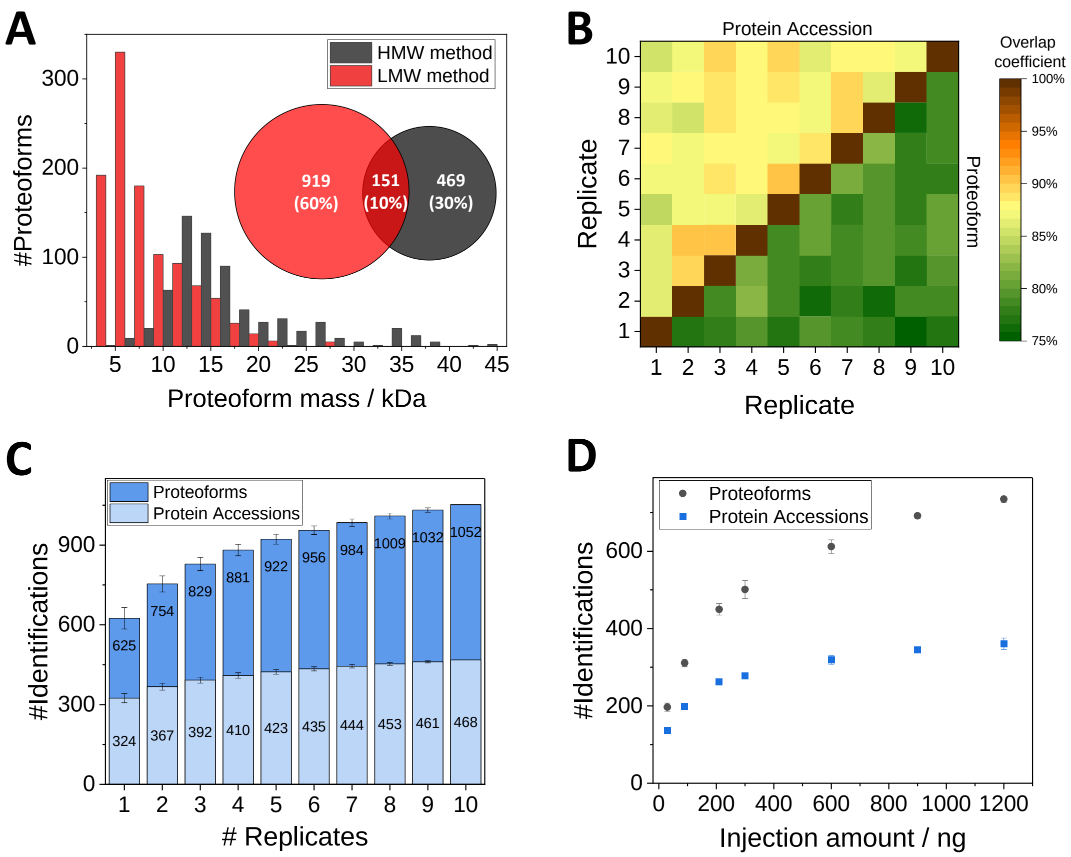

Supplementary Figure 1: **Selection of a suitable LC-FAIMS-MS/MS workflow**. (A) Proteoform mass distribution and proteoform overlap of the two methods utilizing different FAIMS-MS settings to target the high- (HMW) and low/medium-molecular-weight (LMW) proteoforms. (B) Proteoform and protein accession overlap coefficients of technical replicates using the LMW method. (C) Number of identified proteoforms and proteins using the LMW method as a function of the number of technical replicates. All raw files were analyzed together in a multi-consensus analysis, and the number of proteoforms identified in a combination of different numbers of replicates was calculated (average±standard derivation, technical replicates with n = $\binom{\#combined\ replicates}{\#all\ replicates}$). (D) Influence of the injection amount on the number of identifications using the LMW method (n=3 technical replicates, average±standard derivation).

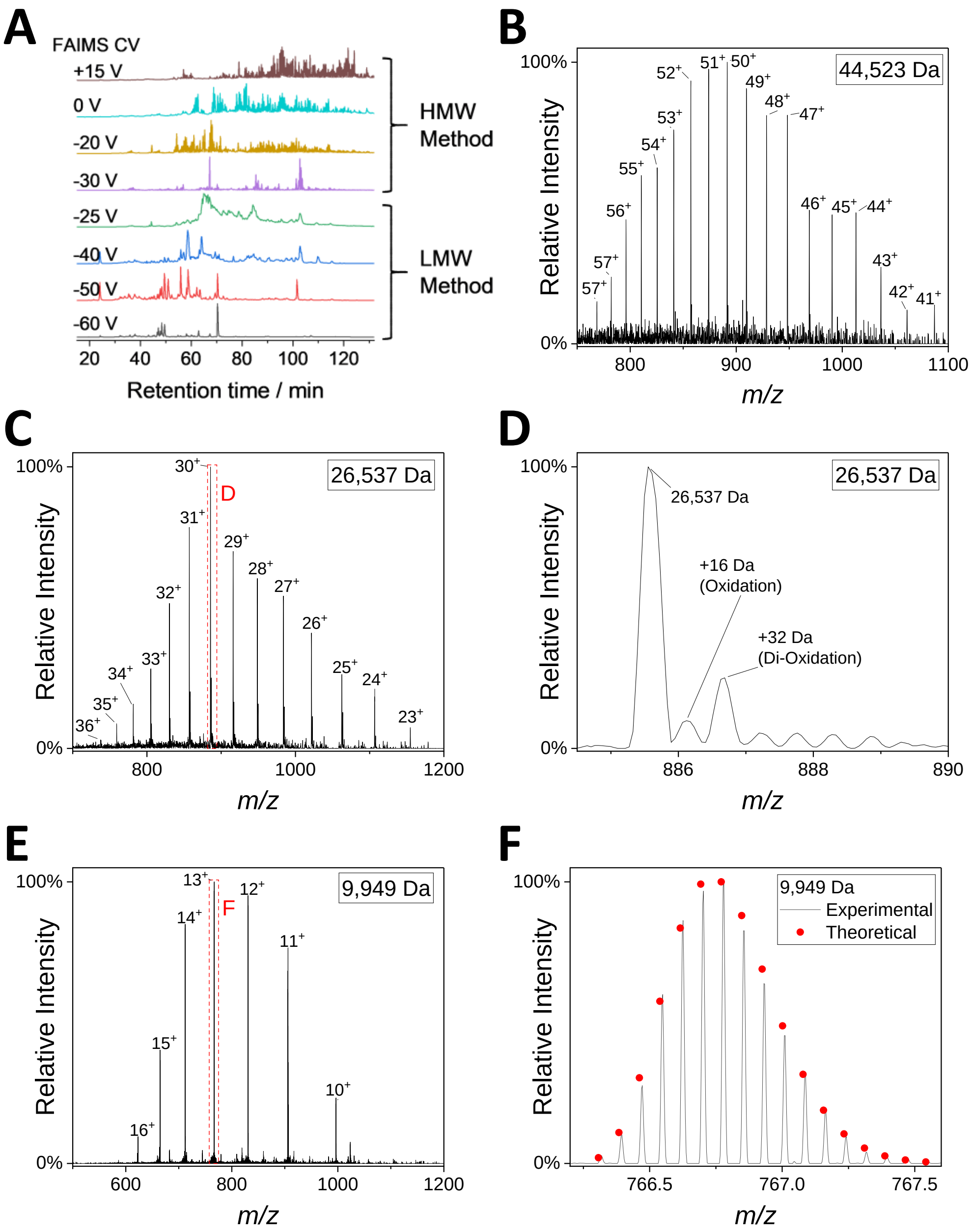

Supplementary Figure 2: **Advantages of the acquisition methods used in this study for proteoform analysis**. Different FAIMS-MS settings were used, targeting high- (HMW) and low/medium-molecular-weight (LMW) proteoforms. (A) Total ion counts (TICs) of the different FAIMS CVs demonstrate the excellent separation performance of the gas-phase fractionation strategy. The HMW method used EThcD fragmentation, resulting in a jagged TIC. (B) Low-resolution spectrum of a 44.6 kDa large proteoform with charge states >50. (C) Low-resolution spectrum of a 26.5 kDa proteoform and (D) zoom-in to one charge state reveals several modified forms. (E) High-resolution spectrum of a 9.9 kDa proteoform and (F) zoom-in to one charge state shows the isotopic pattern.

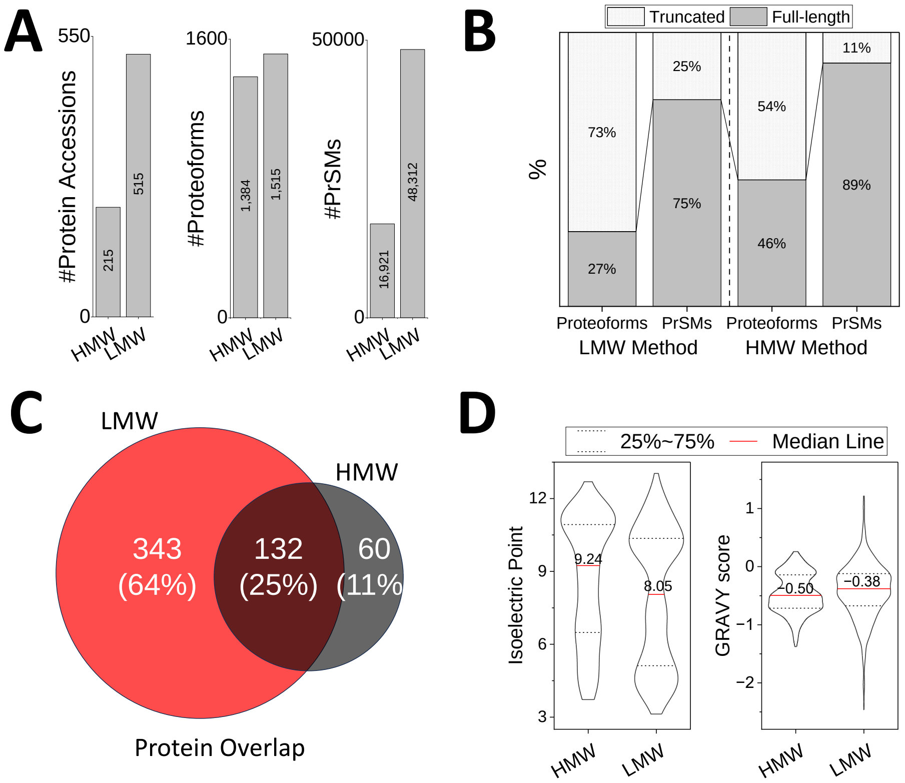

Supplementary Figure 3: **Comparison of the selected LC-FAIMS-MS methods**. (A) Count of identified protein accessions, proteoforms, and proteoform spectral matches (PrSMs). (B) Percentage of full-length and truncated proteoforms and PrSMs assigned to annotated or truncated proteoforms. (C) Overlap of the identified proteins using the LMW (red) and HMW (black) methods. (D) Distribution of the proteoforms' isoelectric points (left) and GRAVY scores (right).

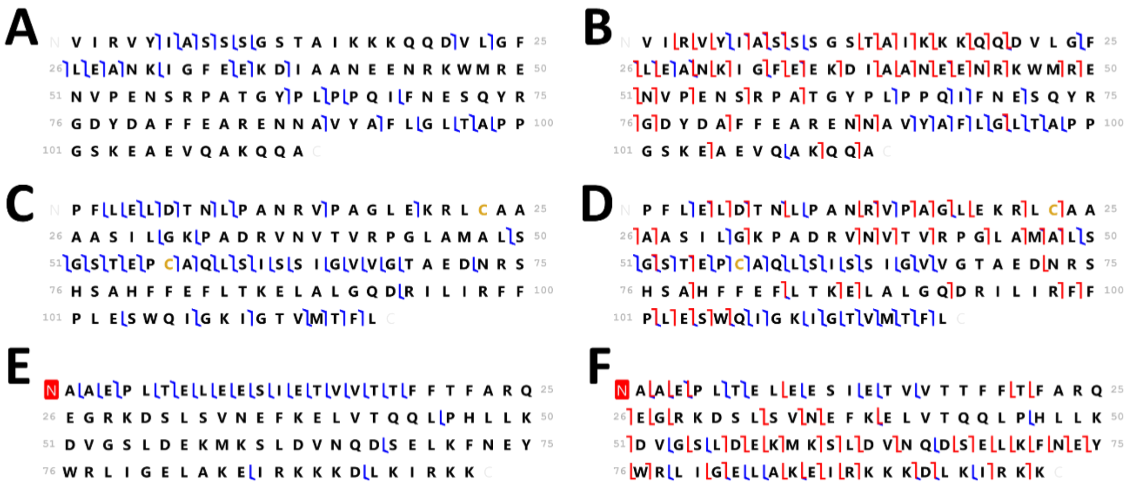

Supplementary Figure 4: **Comparison of the residue cleavage using CID and EThcD fragmentation**. Fragment maps of (A, B) Adapter SH3BGRL (O75368); (C, D) D-dopachrome decarboxylase (P30046); (E, F) Protein S100-A13 (Q99584). CID fragmentation: A, C, E; EThcD fragmentation: B, D, F. Blue brackets represent b/y ions and red brackets c/z ions. For CID fragmentation, b- and y-ions were considered, and for EThcD fragmentation, b-, c-, y-, z-ions.

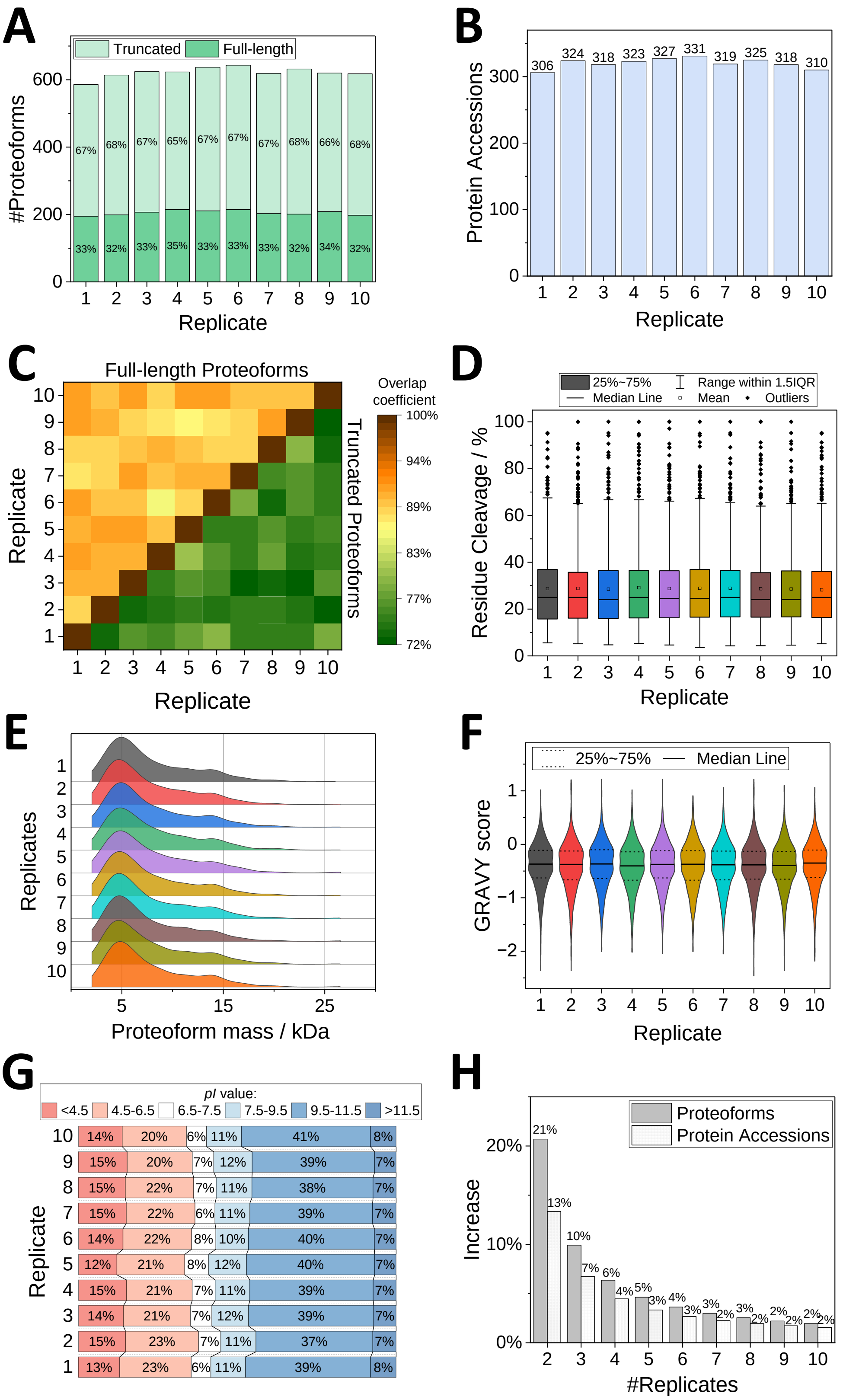

Supplementary Figure 5: **Reproducibility of technical replicates of the LMW method regarding the number and physicochemical properties of the identifications**. (A) Count of identified proteoforms and (B) protein accessions. (C) Overlap coefficients of the identified

full-length and truncated proteoforms. (D) Distribution of the residue cleavage of the identified proteoforms, (E) proteoform mass, (F) GRAVY score, and (G) isoelectric point. The number of data points underlying the distributions correspond to the number of identified proteoforms shown in subfigure A. (H) Percentage increase in the number of proteoform and protein identifications with increasing number of replicates.

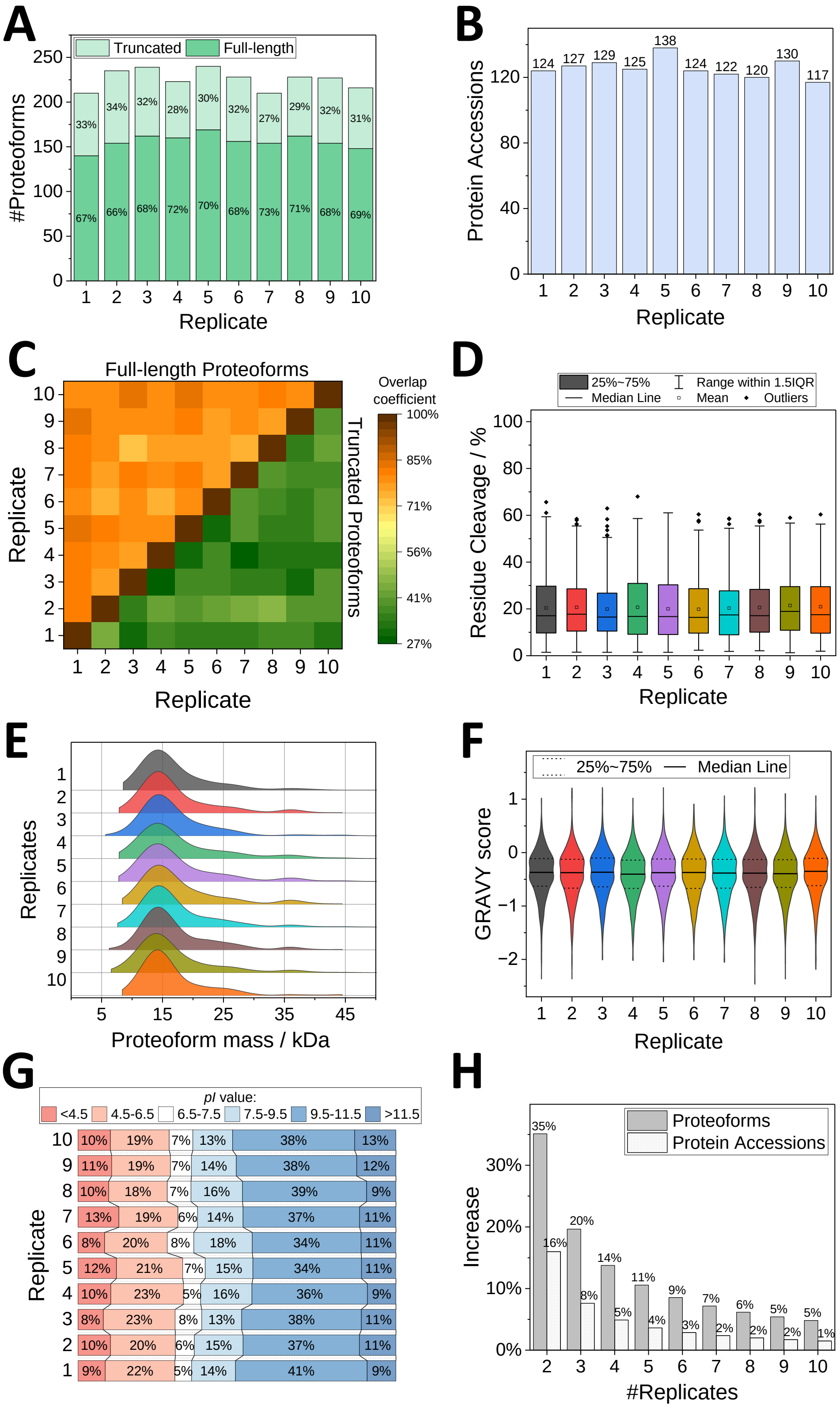

Supplementary Figure 6: **Reproducibility of technical replicates of the HMW method regarding the number and physicochemical properties of the identifications**. (A) Count of identified proteoforms and (B) protein accessions. (C) Overlap coefficients of the identified

full-length and truncated proteoforms. (D) Distribution of the residue cleavage of the identified proteoforms, (E) proteoform mass, (F) GRAVY score, and (G) isoelectric point. The number of data points underlying the distributions correspond to the number of identified proteoforms shown in subfigure A. (H) Percentage increase in the number of proteoform and protein identifications with increasing number of replicates.

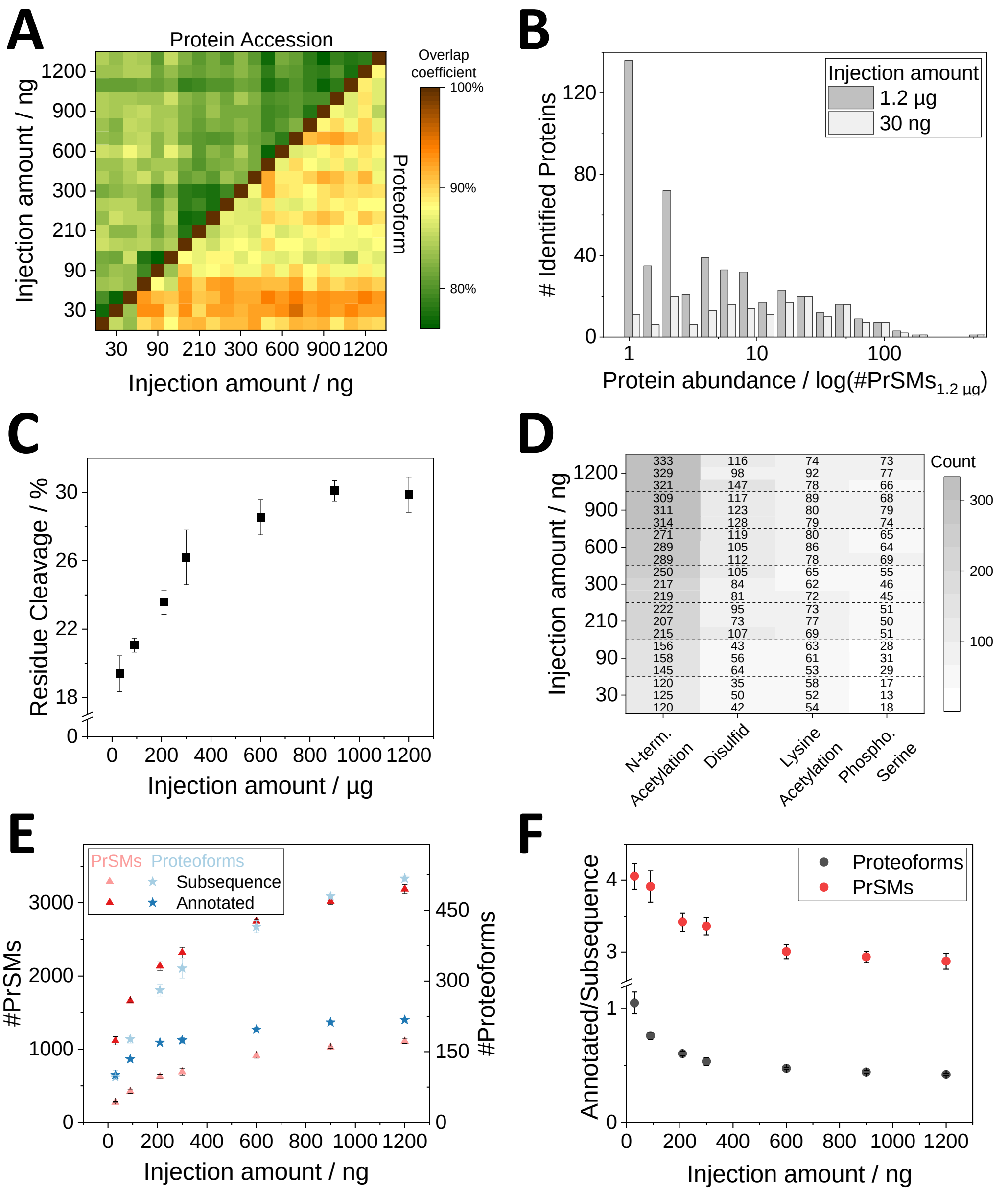

Supplementary Figure 7: **Influence of the amount of injected sample on proteoform and protein identifications**. (A) Overlap coefficients on protein and proteoform levels using various injection amounts. (B) Histogram of the abundance of proteoform identified with 1.2 µg and 30 ng injection. The abundance displayed on the x-axis is based on the logarithmic number of PrSMs identified with 1 1.2 µg injection. (C) Average residue cleavage (n=3 technical replicates, with standard derivation), and (D) number of identified modifications. (E) Average number of PrSMs and proteoforms assigned to annotated and subsequence proteoforms (n=3 technical replicates, average±standard derivation). (F) Ratio of PrSMs and proteoforms assigned to annotated and subsequence proteoforms (n=3 technical replicates, average±standard derivation).

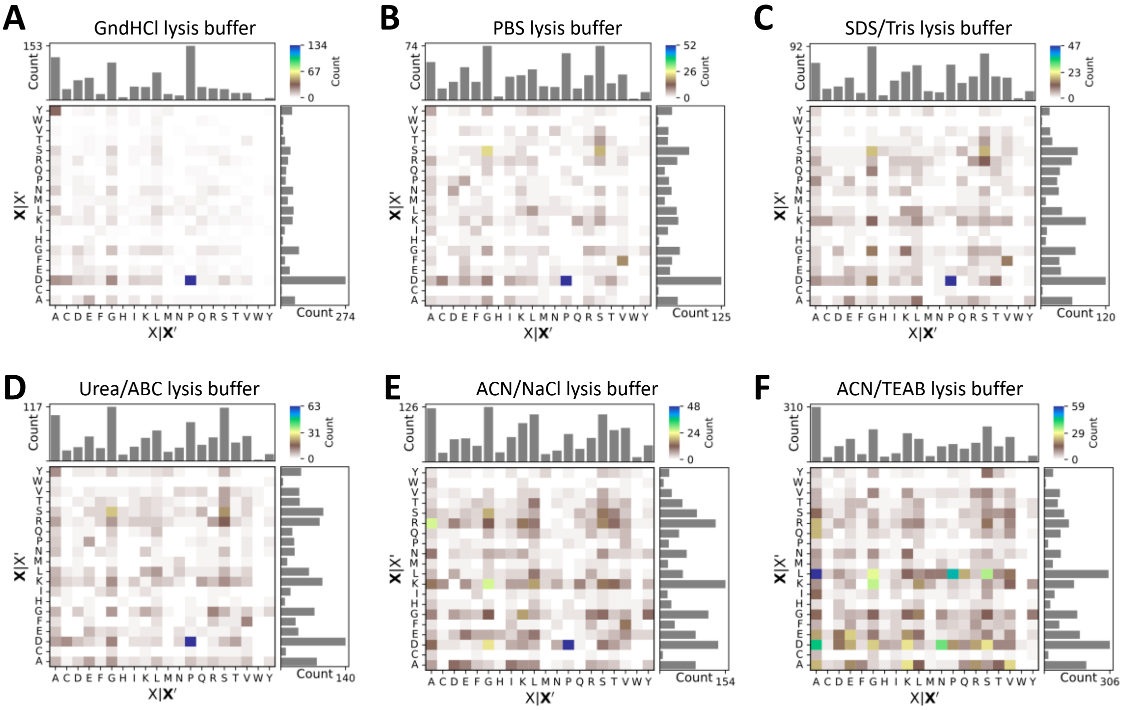

Supplementary Figure 8: **Analysis of identified truncated proteoforms after various cell lysis conditions**. The two-dimensional histograms display the potential cleavage sites of truncated proteoforms identified after lysis with (A) GndHCl, (B) PBS, (C) SDS/Tris, (D) Urea/ABC, (E) ACN/NaCl, and (F) ACN/TEAB. The amino acids N- and C-terminal of the potential cleavage sites are denoted as X and X', respectively.

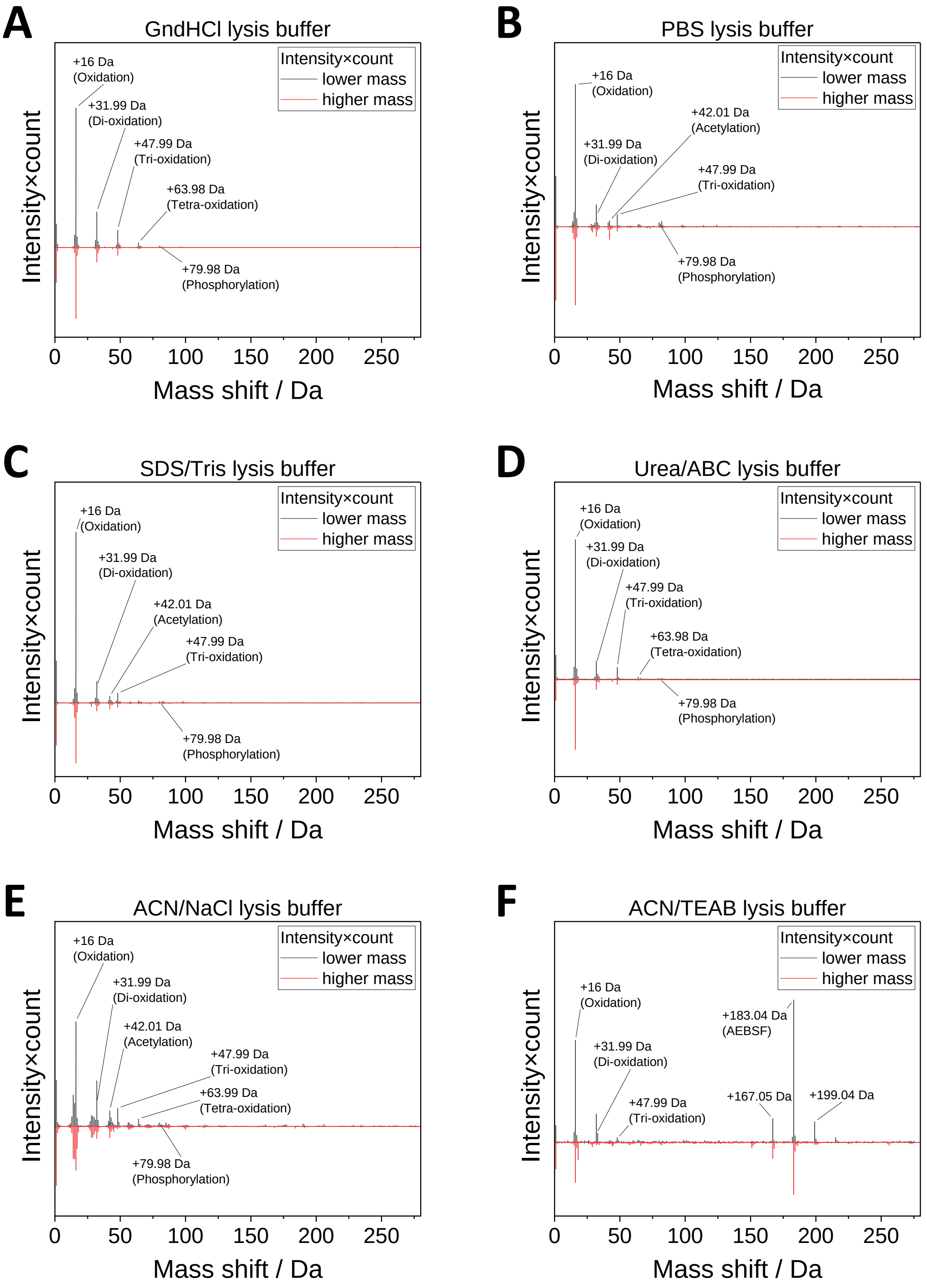

Supplementary Figure 9: **Influence of the cell lysis conditions on the introduction of artificially modified proteoforms or stable non-covalent adduct formation**. Randomly selected raw files for each cell lysis condition were deconvolved with FLASHDeconv and analyzed with MSTopDiff. (A) Intensity×count histogram after lysis with GndHCl, (B) PBS, (C) SDS/Tris, (D) Urea/ABC, (E) ACN/NaCl, and (F) ACN/TEAB. Note that the signals at 14.01 Da and 28.01 Da in (E) derive from unknown contamination (see also Supplementary Notes: Various Observations).

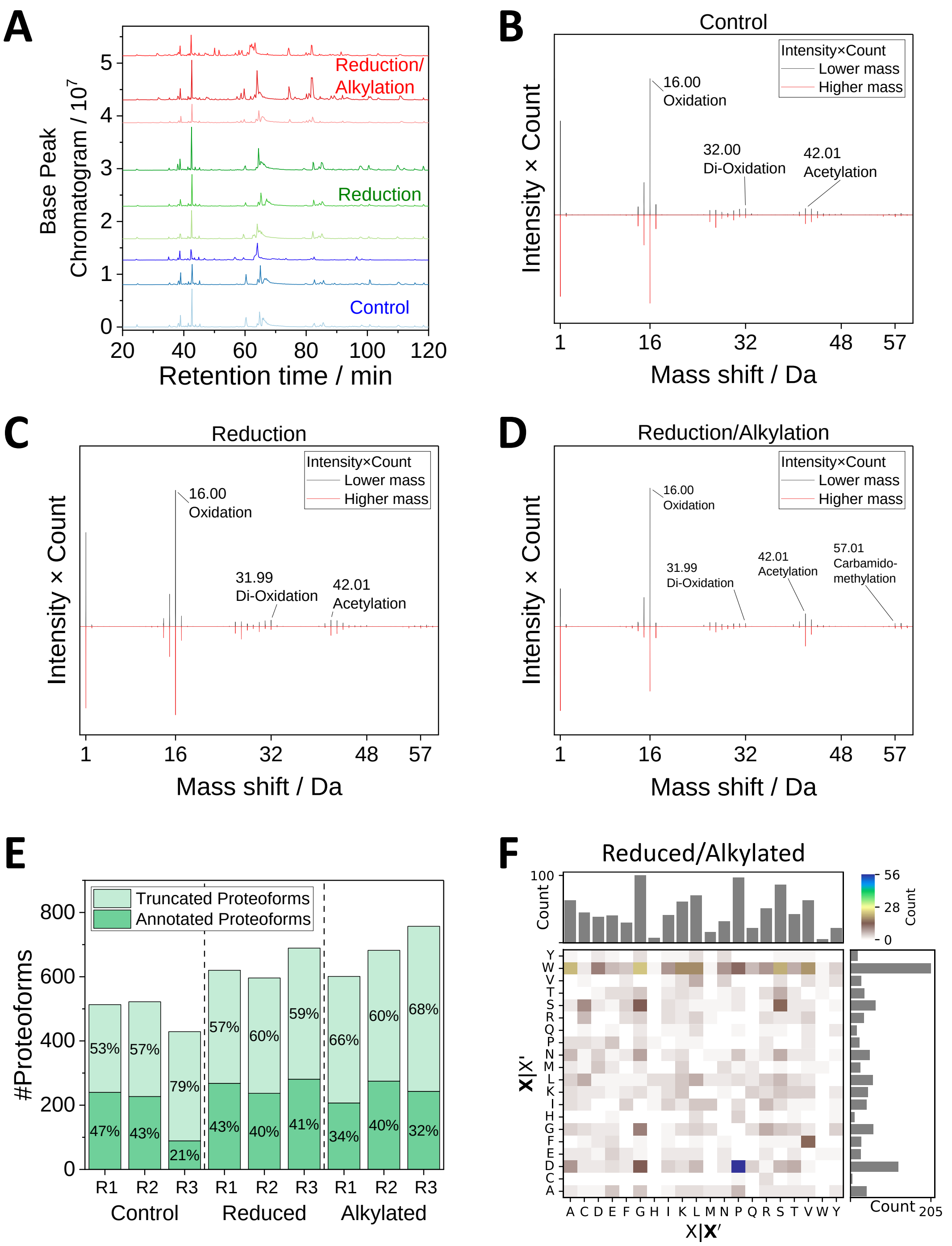

Supplementary Figure 10: **Influence of reduction/alkylation on proteoform identification**. (A) Base peak chromatograms of all replicates (LMW method, CV −40 V). (B) MSTopDiff analysis of randomly selected raw files from the control, (C) reduced, and (D) reduced/alkylated samples. Deconvolution was performed with FLASHDeconv prior to MSTopDiff analysis. (E) Number of truncated and full-length proteoforms. (F) Analysis of identified truncated proteoforms after reduction/alkylation of the sample. The two-dimensional histogram displays the potential cleavage sites of truncated proteoforms. The amino acids N- and C-terminal of the potential cleavage sites are denoted as X and X'.

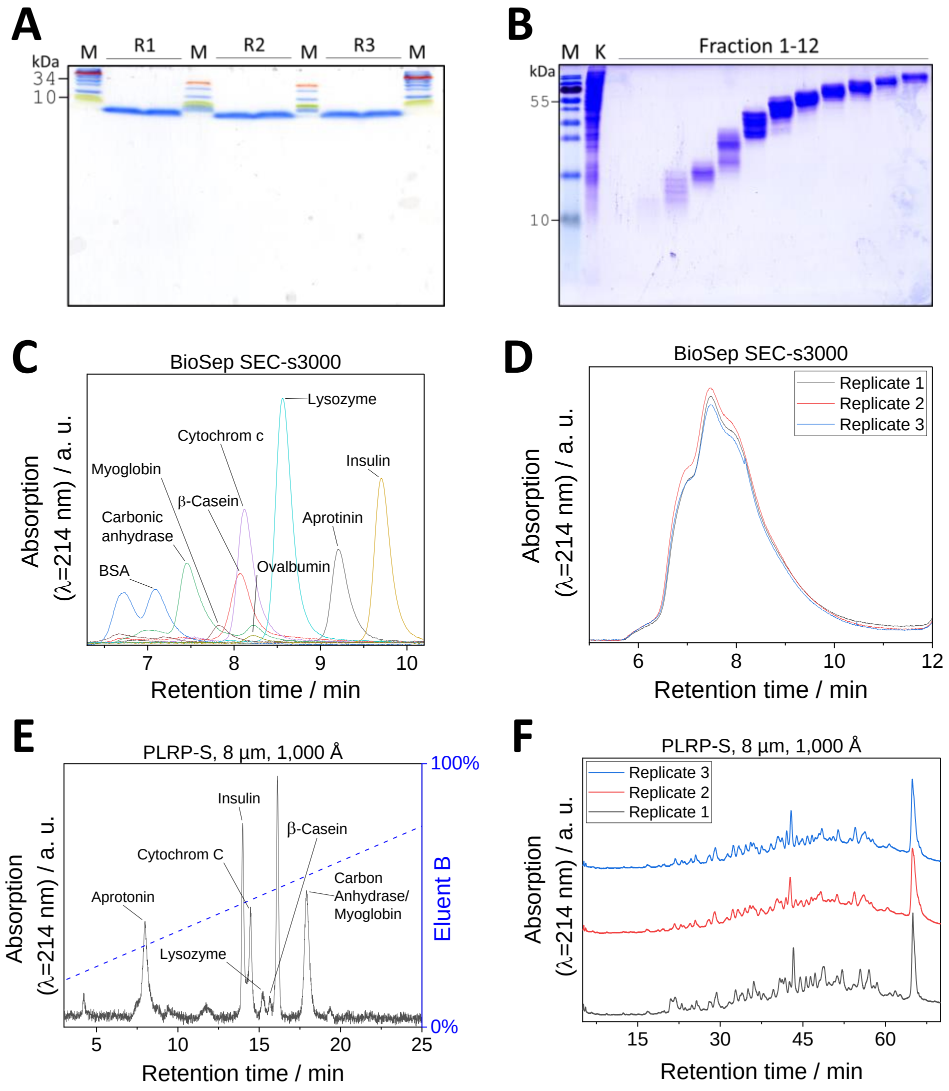

Supplementary Figure 11: **Quality control of the proteoform isolation approaches**. (A) PEPPI approach: Three replicates of Caco-2 proteoforms were separated by SDS-PAGE until proteoforms smaller than 30 kDa (based on a prestained marker) were separated. The gel is shown directly after electrophoresis and before excision of the gel bands below ~30 kDa (M: Protein marker, R1-R3: replicates). (B) GELFrEE approach: After fractionation of the whole proteome, 7.5 µl of each fraction was separated on an SDS-PAGE. The success of all three fractionations was verified by SDS-PAGE analysis, and a representative Coomassie-stained gel of one replicate is shown. (C) SEC approach: Prior to the enrichment of suitable proteoforms, nine proteins were separated to ensure that the column and HPLC were in appropriate condition. The chromatograms (λ=214 nm) from the model proteins and (D) from the full Caco-2 proteome are shown. (E) Low pH fractionation: A protein mixture was separated prior to the fractionation to ensure that the column and HPLC were in appropriate condition. The chromatograms (λ=214 nm) from the six model proteins and (F) from the fractionation of the Caco-2 proteome are shown.

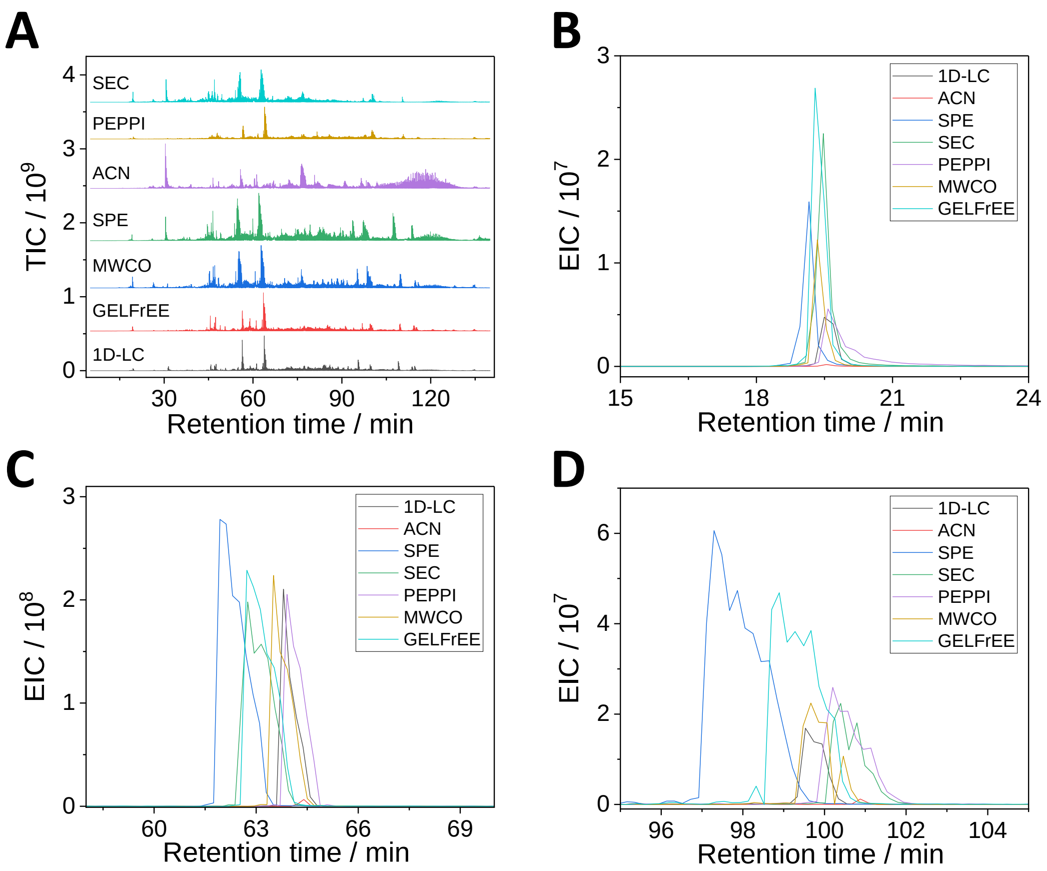

Supplementary Figure 12: **Quality control of the datasets comparing different proteoform isolation strategies**. (A) Total ion chromatograms (TICs) of one representative replicate for each approach. (B) Extracted ion chromatograms of three randomly selected *m/z* values at the start, (C) middle, and (D) end of the gradient. MWCO, 30 kDa filter; ACN, acidic (TFA/NaCl) acetonitrile depletion; SPE, C18 material; PEPPI with subsequent methanol-chloroform-water precipitation.

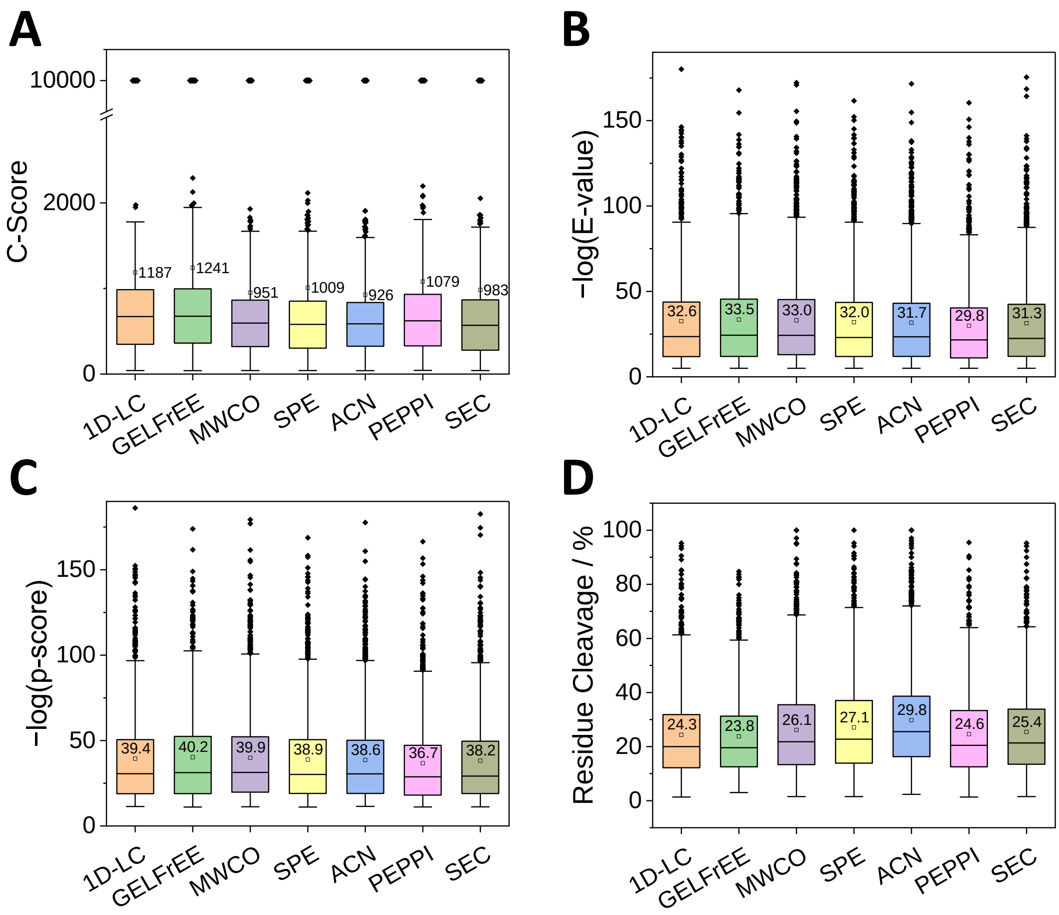

Supplementary Figure 13: **Quality scores of the proteoforms identified using various proteoform isolation strategies**. (A) Distribution of the C-score, (B) −log(*E*-value), (C) −log(*p*-score), and (D) residue cleavage of the identified proteoforms as reported by the database search engine. The boxplots represent the 25-75% interval, with the whiskers within the 1.5 interquartile range and outliers displayed as filled dots. The black lines represent the medians. The unfilled rectangles and the labels show the mean value. The number of data points underlying the box plots corresponds to the number of identified proteoforms in all three replicates (1D-LC: 1,246, GELFrEE: 1,185, MWCO: 1,842, SPE: 1,761, ACN: 2,041, PEPPI: 1,288, SEC: 1,415). MWCO, 30 kDa filter; ACN, acidic (TFA/NaCl) acetonitrile depletion; SPE, C18 material; PEPPI with subsequent methanol-chloroform-water precipitation.

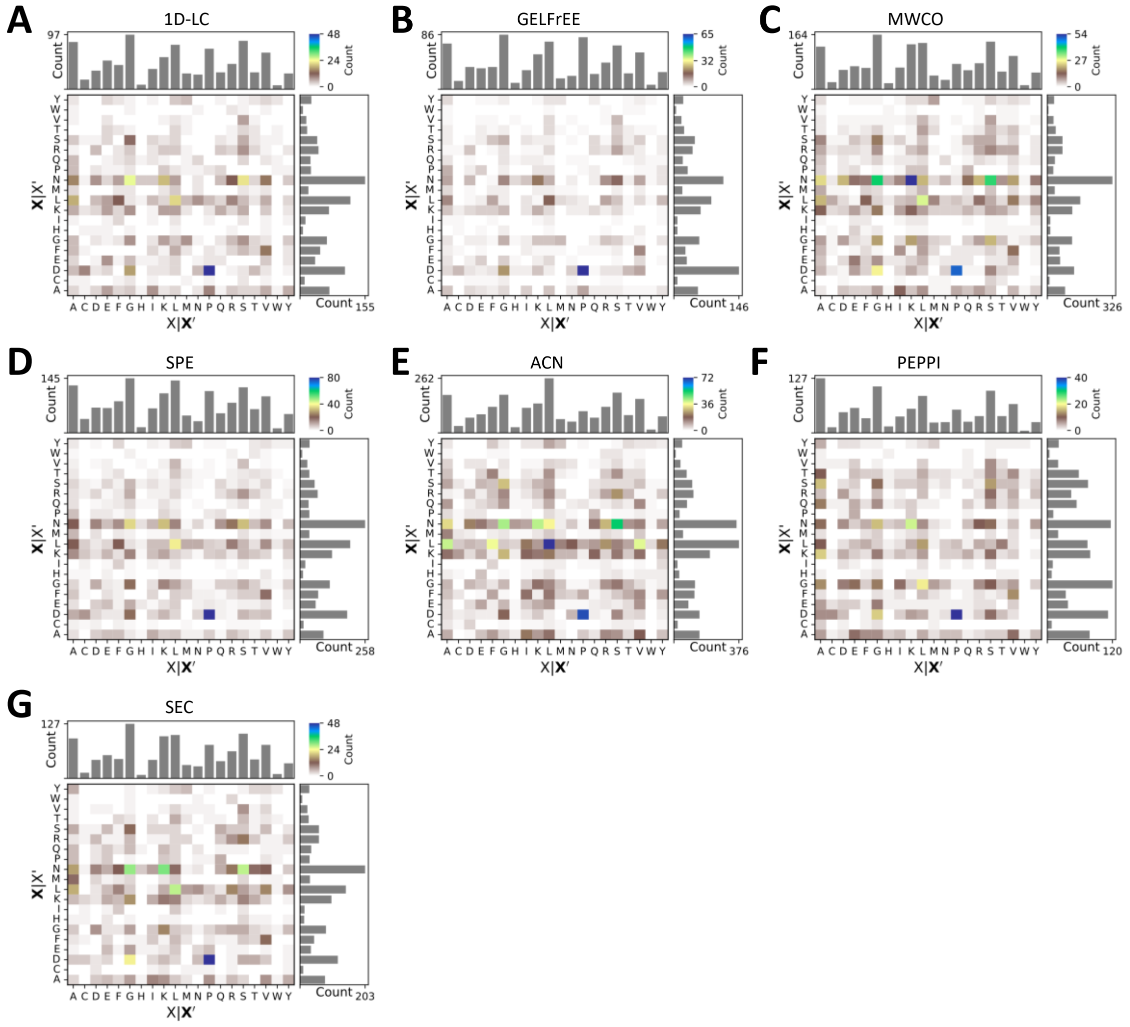

Supplementary Figure 14: **Analysis of identified truncated proteoforms after various proteoform isolation strategies**. Two-dimensional histograms displaying the preceding (X) and subsequent (X') amino acids of truncated proteoforms (A) without sample preparation (1D-LC), after sample preparation using (B) GELFrEE, (C) MWCO filter (30 kDa), (D) SPE (C18 material), (E) acidic (TFA/NaCl) acetonitrile depletion, (F) PEPPI with subsequent methanol-chloroform-water precipitation, and (G) SEC.

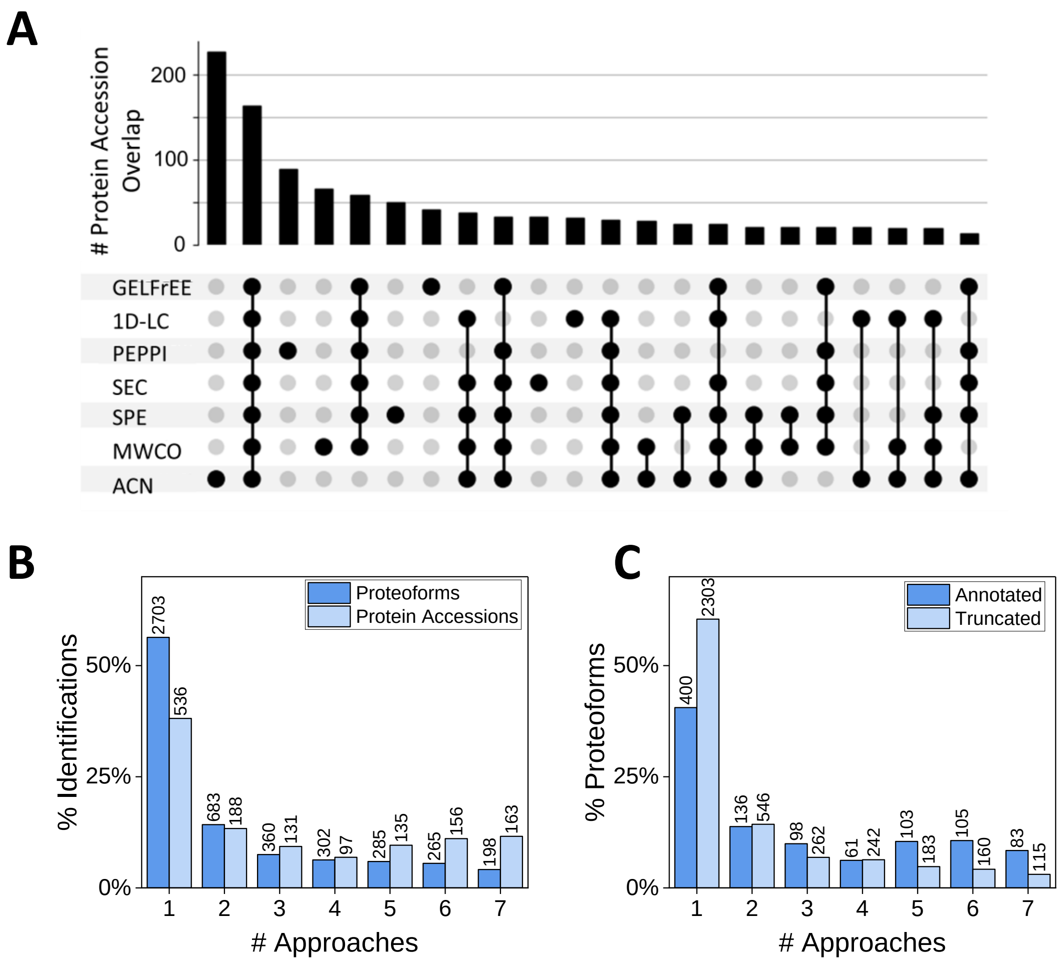

Supplementary Figure 15: **Overlap and complementarity of various sample preparation strategies**. (A) UpSet plot of protein accessions identified with the various approaches.[38] (B) Total count (labels) and percentage of proteoforms and proteins and (C) truncated and annotated proteoforms identified in one or more sample preparation strategies. MWCO, 30 kDa filter; ACN, acidic (TFA/NaCl) acetonitrile depletion; SPE, C18 material; PEPPI with subsequent methanol-chloroform-water precipitation.

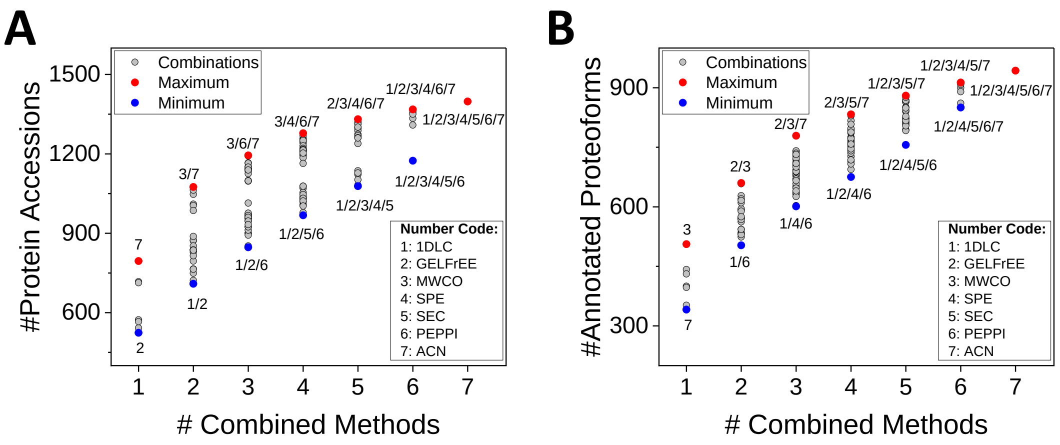

Supplementary Figure 16: **Influence of the combination of different proteoform isolation strategies**. Number of identified (A) protein accessions and (B) annotated (full-length) proteoforms. MWCO, 30 kDa filter; ACN, acidic (TFA/NaCl) acetonitrile depletion; SPE, C18 material; PEPPI with subsequent methanol-chloroform-water precipitation.

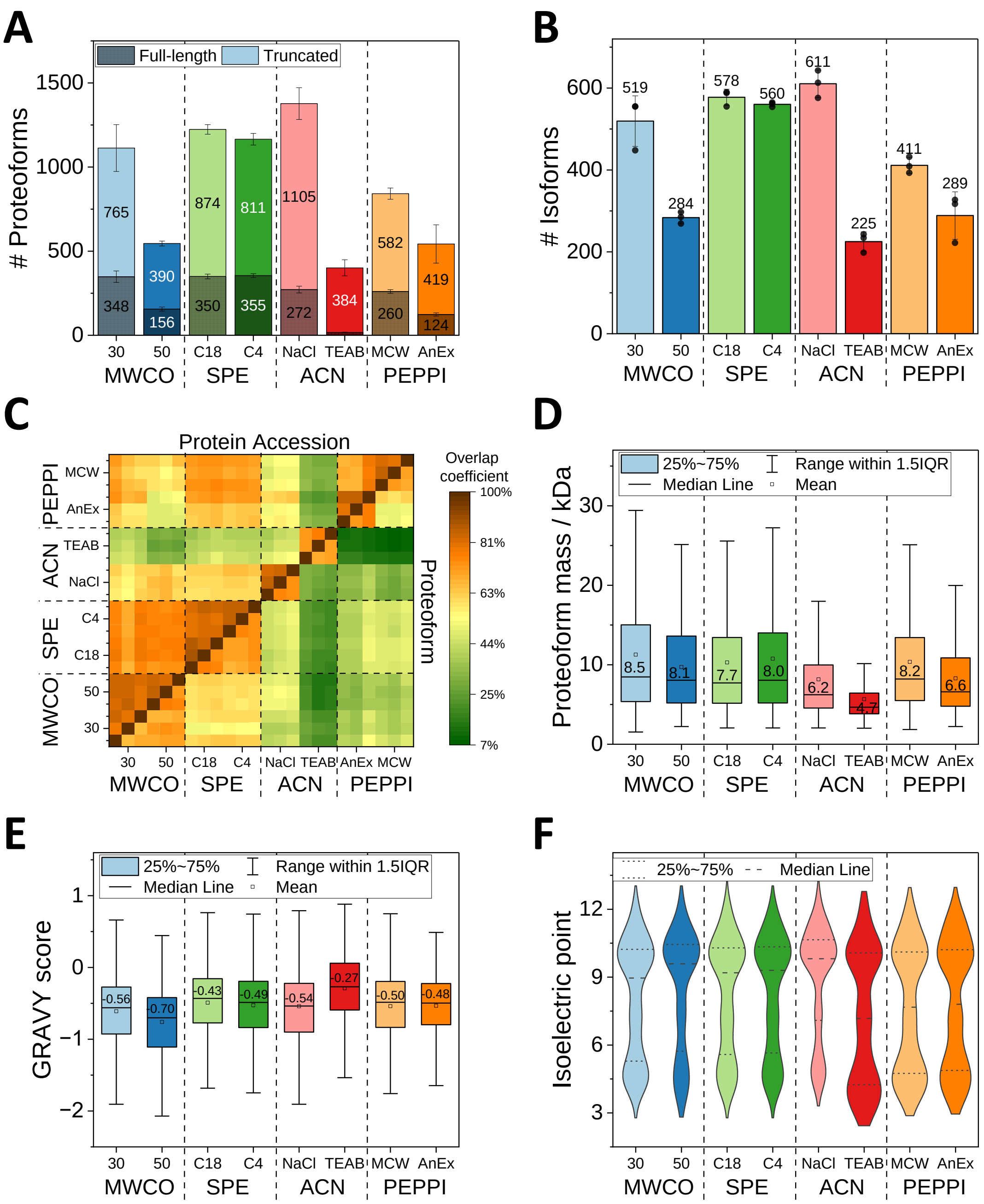

Supplementary Figure 17: **Influence of variations of sample preparation strategies on proteoform identifications**. (A) Number of identified proteoforms and (B) protein acccessions (n=3 replicates of independently performed sample preparations, average±standard derivation). (C) Overlap coefficients on protein and proteoform levels. (D) Distribution of the proteoform mass, (E) GRAVY score, and (F) isoelectric point. The labels represent the mean value. The number of data points underlying the distributions corresponds to the number of identified proteoforms after combining all three replicates (MWCO 30 kDa: 1,842, MWCO 50 kDa: 793, SPE C18: 1,761, SPE C4: 1,689, ACN NaCl: 2,041, ACN TEAB: 631, PEPPI-MCW: 1,288, PEPPI-AnExSP: 858).

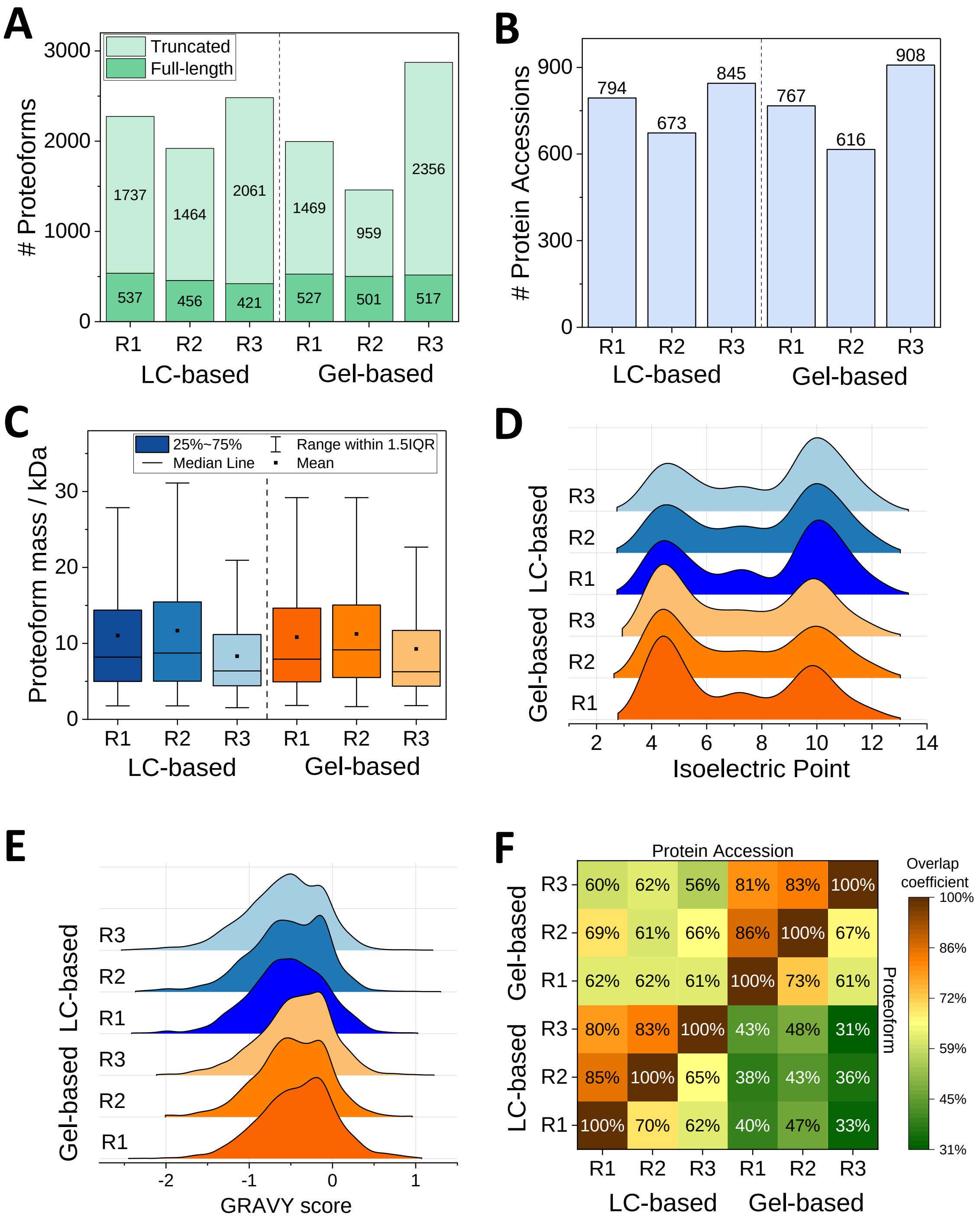

Supplementary Figure 18: **Influence of multidimensional separation schemes on the identification of proteoforms and proteins**. (A) Number of identified full-length and truncated proteoforms and (B) protein accessions. The label shows the average (n=3). Distribution of the proteoform (C) mass, (D) isoelectric point (*pI*), and (E) GRAVY score. The number of data points underlying the distributions corresponds to the number of identified proteoforms shown in subfigure A. (F) Overlap coefficients between the replicates of the multidimensional separation schemes regarding the identified proteins and proteoforms.

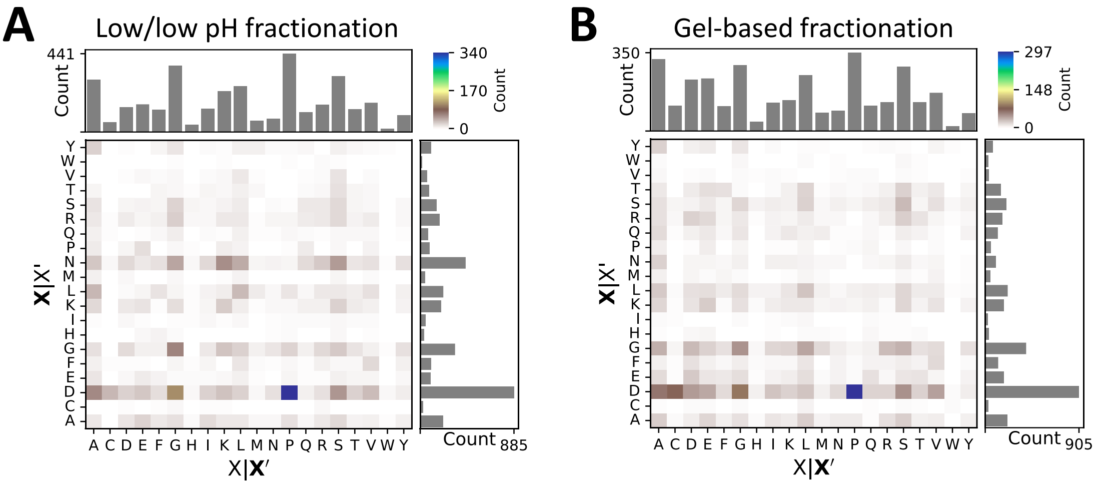

Supplementary Figure 19: **Analysis of identified truncated proteoforms after multidimensional separation schemes**. Two-dimensional histograms displaying the preceding (X) and subsequent (X') amino acids of truncated proteoforms after (A) low pH fractionation and (B) GELFrEE fractionation.

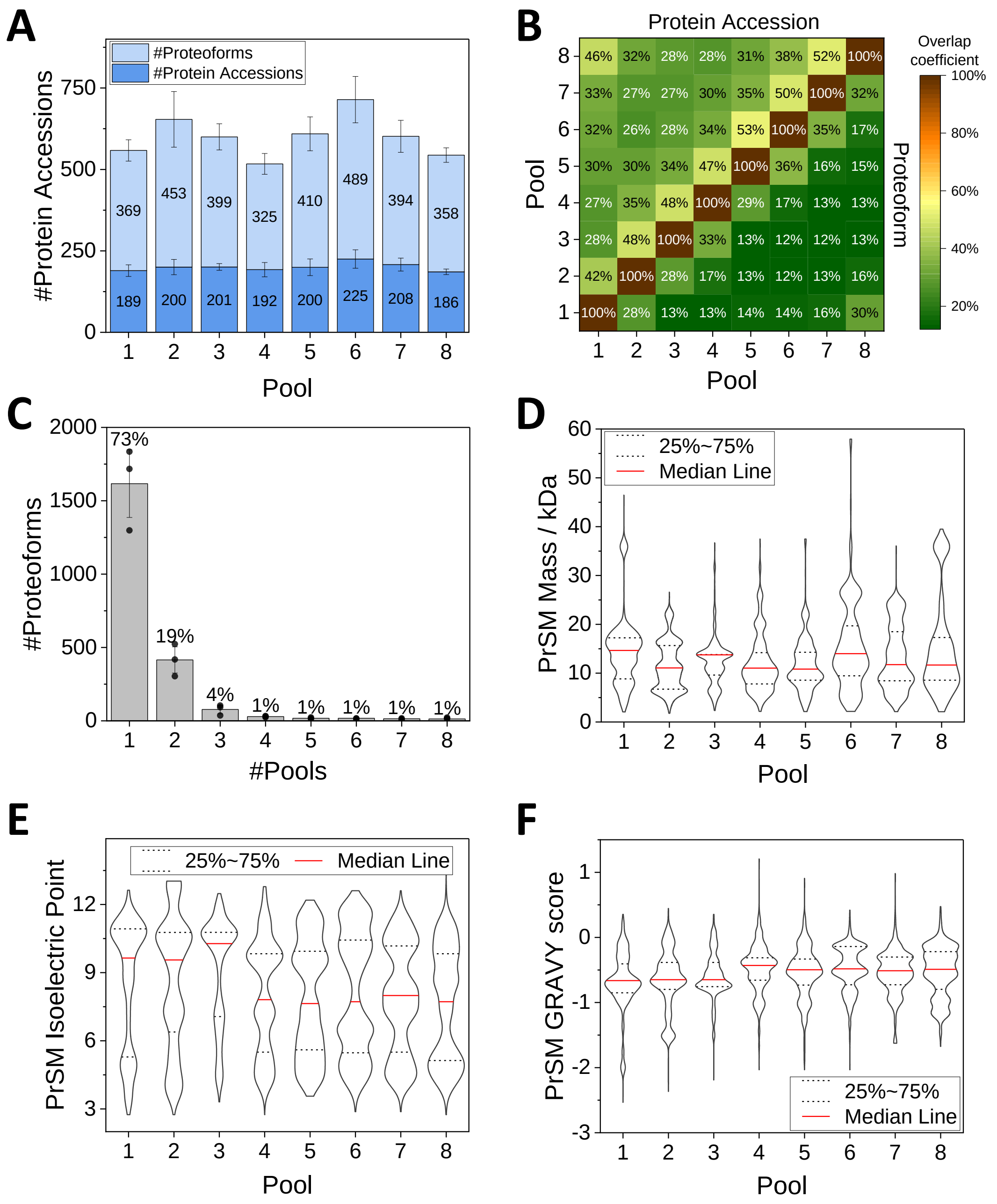

Supplementary Figure 20: **Fractionation efficiency of the low/low pH reversed-phase chromatography separation scheme**. (A) Number of identified proteoforms and protein accessions (n=3 replicates of independently performed sample preparations, average±standard derivation) and (B) overlap coefficient of the various pools. (C) Number of proteoforms that were identified exclusively in one to eight pools (n=3 replicates of independently performed sample preparations, average±standard derivation). (D) Distribution of the mass, (E) isoelectric point, and (F) GRAVY score of the identified proteoform spectral matches (PrSMs) in the different pools.

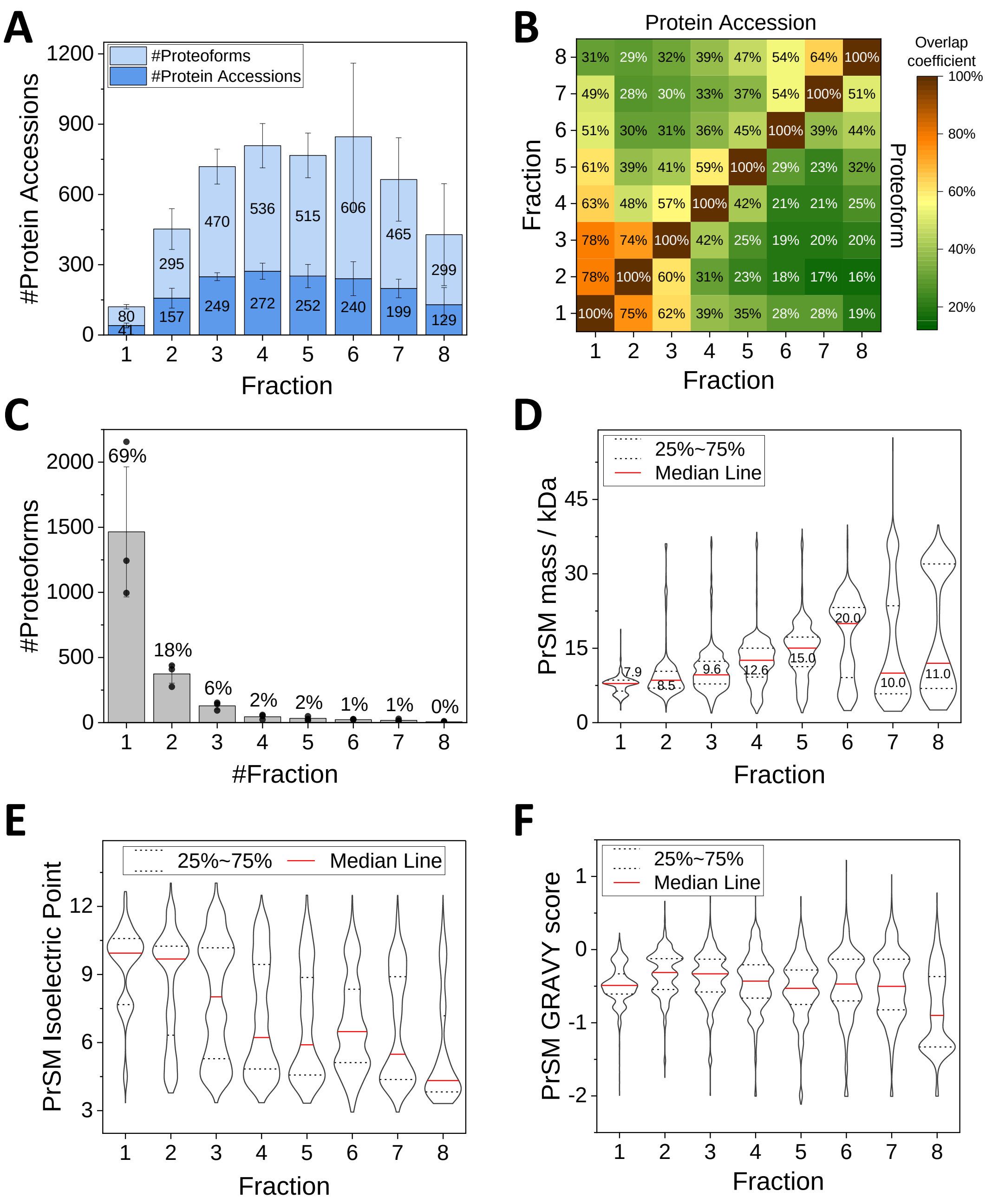

Supplementary Figure 21: **Fractionation efficiency of the gel-based separation scheme**. (A) Number of identified proteoforms and protein accessions (n=3 replicates of independently performed sample preparations, average±standard derivation) and (B) overlap coefficient of the various pools. (C) Number of proteoforms that were identified exclusively in one to eight fractions (n=3 replicates of independently performed sample preparations, average±standard derivation). Distribution of the (D) mass, (E) isoelectric point, and (F) GRAVY score of the identified proteoform spectral matches (PrSMs) in the different pools.

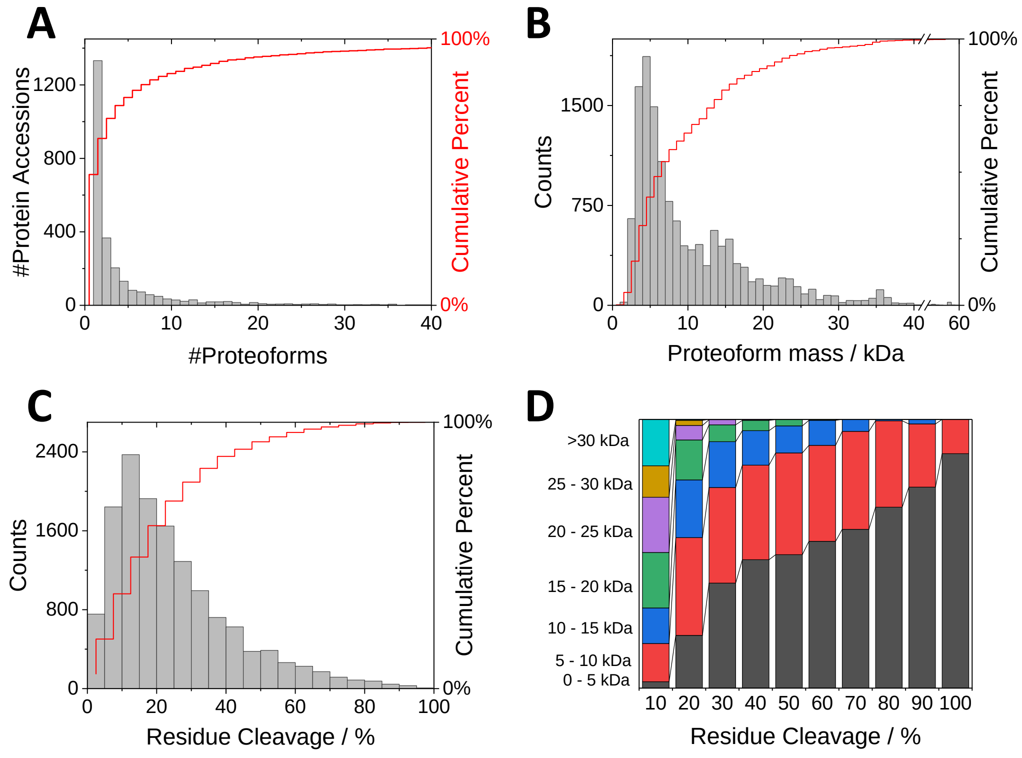

Supplementary Figure 22: **Properties of proteoforms identified in this study**. (A) Histogram of the protein accessions regarding the number of associated proteoforms. (B) Distribution of proteoform masses and (C) obtained residue cleavage. (D) Residue cleavage depending on the proteoform mass.

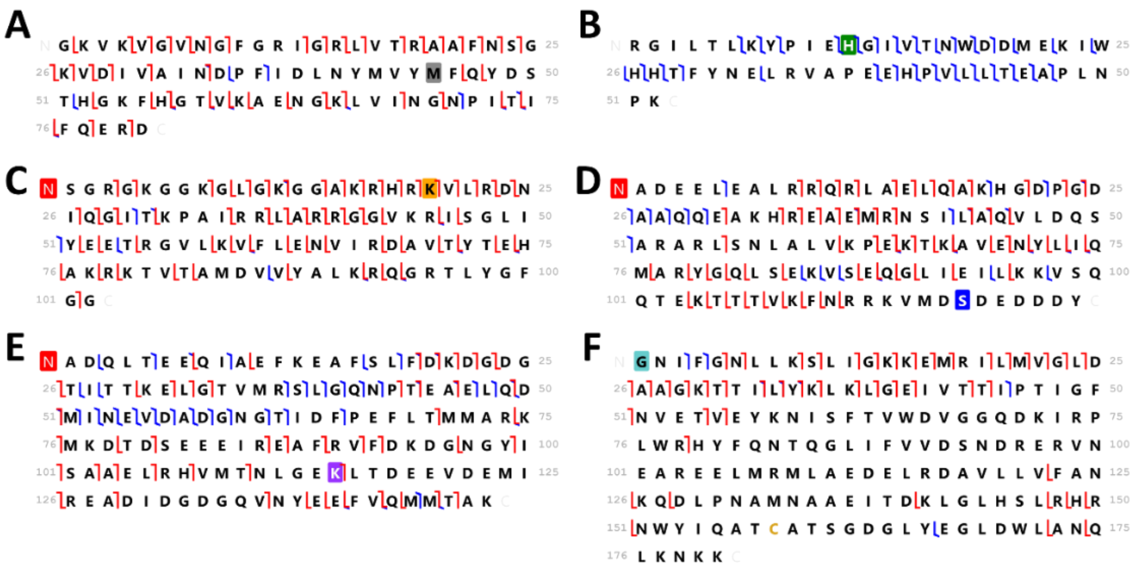

Supplementary Figure 23: **Selected proteoforms carrying various PTMs**. Fragment maps of proteoforms from (A) Glyceraldehyde-3-phosphate dehydrogenase (P04406), (B) Actin (P60709), (C) Histon H4 (P62805), (D) Programmed cell death protein 5 (O14737), (E) Calmodulin (P0DP23), (F) ADP-ribosylation factor 3 (P61204). Highlighted in: grey, methionine oxidation; red, N-terminal acetylation; green, histidine methylation; orange, lysine butyrylation; blue, serine phosphorylation; purple, tri-methylation; blue-green, N-terminal myristoylation. EThcD fragmentation: A, C, D, E, F; CID fragmentation: B. Blue brackets represent b/y ions and red brackets c/z ions. For CID fragmentation, b- and y-ions were considered, and for EThcD fragmentation, b-, c-, y-, z-ions.

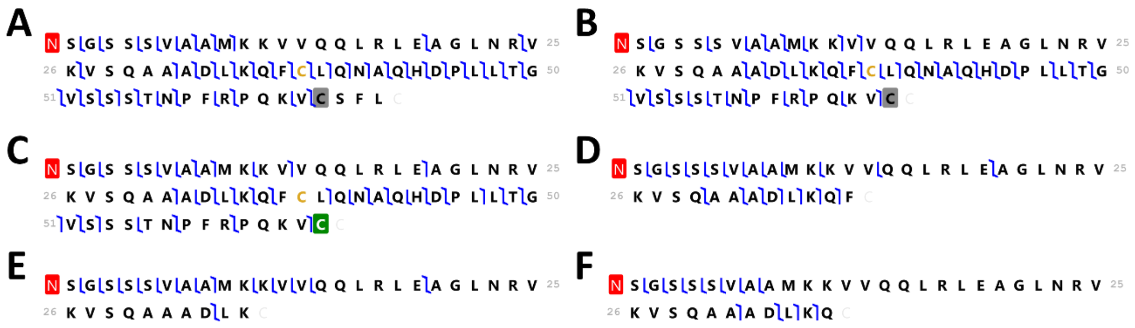

Supplementary Figure 24: **Different proteoforms of the guanine nucleotide-binding protein G(I)/G(S)/G(O) subunit gamma-5 (P63218)**. Fragment maps after CID fragmentation of proteoforms with (A) S-geranylgeranylation cysteine, (B) S-geranylgeranylation cysteine and cleaved propeptide, (C) cysteine methyl ester and cleaved propeptide, and (D-F) previously undescribed C-terminal truncations. Highlighted in: red, N-terminal acetylation; grey: S-geranylgeranyl cysteine; green, cysteine methyl ester. Blue brackets represent b/y ions. For CID fragmentation, b- and y-ions were considered.

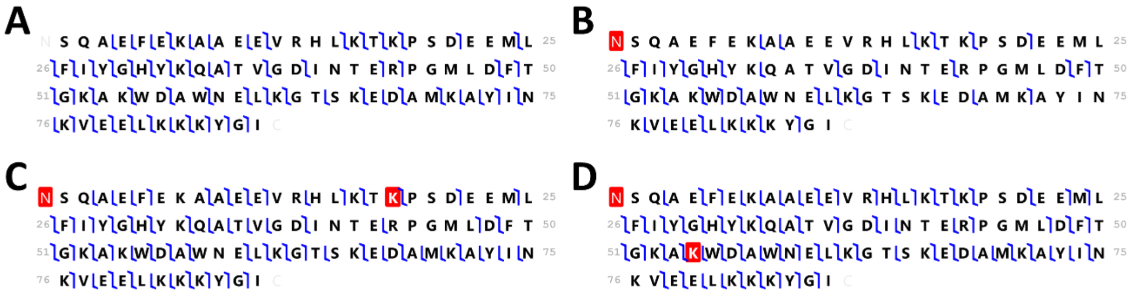

Supplementary Figure 25: **Different proteoforms of the acyl-CoA-binding protein (P07108)**. Fragment maps after CID fragmentation of the (A) unmodified, (B) N-terminal acetylated, (C) N-terminal and K19 acetylated, and (D) N-terminal acetylated and K55 acetylated form. Blue brackets represent b/y ions. For CID fragmentation, b- and y-ions were considered.

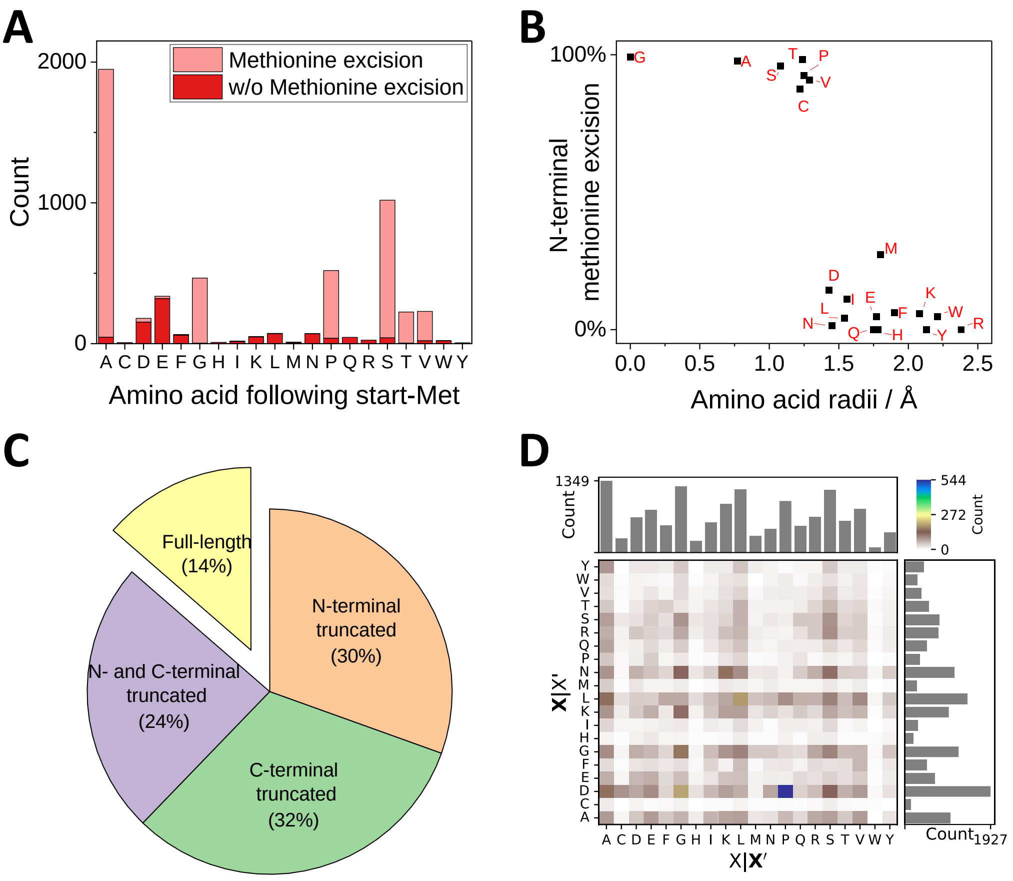

Supplementary Figure 26: **Identified proteoform termini in this study**. (A) Count of the start-methionine excision and (B) ratio of start-methionine excision depending on the amino acids radii of gyration. (C) Percentage of full-length and truncated proteoforms. (D) Two-dimensional histograms displaying the preceding (X) and subsequent (X') amino acids of truncated proteoforms.

Supplementary Tables

Supplementary Table 1: **Overview of LC-MS settings utilized in this study**. LMW method: targeting low/medium-molecular-weight-range proteoforms <20 kDa. HMW method: targeting high-molecular-weight-range proteoforms >20 kDa.

Setting	LMW method	HMW method
**CVs**	−60, −50, −40, −25 V	−30, −20, 0, +15 V
**Acquisition mode**	High/High	Medium/High
**Pressure mode (IRM)**	Peptide mode (8 mTorr)	Protein mode (2 mTorr)
**Fragmentation**	CID, 25%	EThcD, 10 ms ETD, 25% HCD
**Cycle time**	3 s	3 s
**Dynamic Exclusion**	Two times within 30 s, 60 s exclusion, ±1.5 *m/z* tolerance	Two times within 30 s, 60 s exclusion, ±1.5 *m/z* tolerance
**Mass range MS1**	400-1,800 *m/z*	400-1,800 *m/z*
**Mass rage MS2**	150-2,000 *m/z*	Automatic
**Resolution MS1**	CV −60, −50 V: 60,000<br>CV −40, −25 V: 120,000	7,500
**Resolution MS2**	CV −60, −50 V: 50,000<br>CV −40, −25 V: 60,000	60,000
**AGC target MS1**	200%	200%
**AGC target MS2**	CV −60, −50 V: 800%<br>CV −40, −25 V: 1,000%	1,000%
**Maximal injection time MS1**	CV −60, −50 V: 118 ms<br>CV −40, −25 V: 246 ms	50 ms
**Maximal injection time MS2**	CV −60, −50 V: 200 ms<br>CV −40, −25 V: 250 ms	250 ms
**# Microscans MS1**	CV −60, −50 V: 2<br>CV −40, −25 V: 4	10
**# Microscans MS2**	CV −60, −50 V: 2<br>CV −40, −25 V: 4	6

Supplementary Table 2: **Number of assigned modifications depending on cell lysis conditions**.

Modification	ACN/NaCl	ACN/TEAB	PBS	GndHCl	SDS/Tris	Urea/ABC
alpha-amino acetylated residue	535	332	452	357	414	450
N6-acetyl-L-lysine	99	56	161	121	105	162
O-phospho-L-serine	166	43	144	115	112	122
half cystine	116	98	73	70	96	80
N6-succinyl-L-lysine	36	18	56	77	59	85
omega-N-methyl-L-arginine	25	1	64	33	48	56
O-phospho-L-threonine	63	8	54	22	30	37
L-citrulline	46		57	4	36	24
asymmetric dimethyl-L-arginine	45		47		32	13
N6-crotonyl-L-lysine	25		45	3	37	22
O4'-phospho-L-tyrosine	11	5	32	24	14	27
symmetric dimethyl-L-arginine	32	2	24	6	22	22
N-myristoylglycine	31	8	5	15	11	8
N6,N6,N6-trimethyl-L-lysine	17	5	14	1	10	1
N6,N6-dimethyl-L-lysine	11		18	3	9	3
N,N,N-trimethyl-L-alanine	5		8	6	9	16
L-allysine	12		20		5	5
N6-methyl-L-lysine	4	3	13	4	6	4
S-palmitoyl-L-cysteine	3		5	10	9	5
L-methionine sulfoxide			9	11	5	5
N6-butanoyl-L-lysine	5		10	1	6	7
O-(ADP-ribosyl)-L-serine			7		12	6
hypusine	3		4	3	6	8
deamidated L-asparagine			7	10	3	4
1'-methyl-L-histidine	10	8				
S-geranylgeranyl-L-cysteine	1	2	1	6	1	7
3'-nitro-L-tyrosine			6	4	4	4
L-cysteine sulfinic acid			6	2	2	1
3-hydroxy-L-proline			3	2	1	2
N6-malonyl-L-lysine	1	2		1	1	2
L-cysteine methyl ester	1	1		1	1	2
S-nitrosyl-L-cysteine			2	1	1	2
L-cysteic acid (L-cysteine sulfonic acid)			1	2	3	
L-methionine (R)-sulfoxide				3		2
pyruvic acid (Ser)	1	1			1	1
N6-lipoyl-L-lysine		1	1			2
3-hydroxy-L-histidine			1	1	1	
N5-methyl-L-glutamine			1		2	
4-hydroxy-L-proline			1			1
dimethylated L-arginine				1		1
N,N,N-trimethylglycine	1					
L-phenylalanine amide					1	

Supplementary Table 3: **Number of assigned modifications depending on the proteoform isolation method.** MWCO, 30 kDa filter; ACN, acidic (TFA/NaCl) acetonitrile depletion; SPE, C18 material; PEPPI with subsequent methanol-chloroform-water precipitation.

Modification	1D-LC	GELFrEE	MWCO	SPE	ACN	PEPPI	SEC
alpha-amino acetylated residue	519	455	705	664	619	506	518
O-phospho-L-serine	137	100	178	153	84	105	173
N6-acetyl-L-lysine	122	103	152	119	75	72	115
half cystine	78	18	156	102	111	41	95
N6-succinyl-L-lysine	48	59	44	38	53	35	43
omega-N-methyl-L-arginine	44	26	57	41	17	44	42
O-phospho-L-threonine	23	28	44	38	39	22	31
O4'-phospho-L-tyrosine	20	23	38	26	13	20	18
symmetric dimethyl-L-arginine	8	11	23	10	39	10	26
N-myristoylglycine	9	6	19	22	12	4	14
L-citrulline	7	13	13	10	15	11	16
N6,N6,N6-trimethyl-L-lysine	5	10	13	15	2	12	9
N,N,N-trimethyl-L-alanine	7	9	14	9	6	13	8
S-palmitoyl-L-cysteine	9	13	11	17	1	3	9
N6-methyl-L-lysine	5	10	9	8	1	6	17
N6-crotonyl-L-lysine	5	6	9	6	8	6	10
asymmetric dimethyl-L-arginine	7	3	7	6	3	4	11
N6-butanoyl-L-lysine	2	8	3	5	4	5	8
1'-methyl-L-histidine	1		5	6	3	14	3
3'-nitro-L-tyrosine	8	2	7	4		4	4
S-geranylgeranyl-L-cysteine	3	5		7	11	1	
N6,N6-dimethyl-L-lysine	5	2	4	8		4	2
L-cysteine sulfinic acid	2	5	10	2		1	4
hypusine	3	3	3	4		5	4
L-methionine sulfoxide	5		3	7		2	
L-methionine (R)-sulfoxide				2		12	2
S-nitrosyl-L-cysteine	2	1	5	2		2	
N,N,N-trimethylglycine	2	2	2	2		1	2
deamidated L-asparagine	3		1	6		1	
O-(ADP-ribosyl)-L-serine		3	3			1	3
L-cysteic acid (L-cysteine sulfonic acid)			7				1
3-hydroxy-L-histidine		1		1			5
N6-lipoyl-L-lysine	1	1	1	1		1	1
O-phosphopantetheine-L-serine		1	1		1	1	
L-phenylalanine amide	1			2			
3-hydroxy-L-proline		1					2
N6-malonyl-L-lysine					1		2
L-cysteine methyl ester	1				1		
S-farnesyl-L-cysteine		1					
N5-methyl-L-glutamine			1				

Supplementary Table 4: **Proteins identified in the study (xlsx-file)**. All database search results were combined using a multi-consensus search.

Supplementary Table 5: **Proteoforms identified in the study (xlsx-file)**. All database search results were combined using a multi-consensus search.

Supplementary Table 6: Proteins with associated proteoforms identified in the study (xlsx-file). All database search results were combined using a multi-consensus search.

Supplementary Table 7: **Overview of different sample preparation steps and their influence on identifying proteoforms by TDP**.

Step	Variations/ Details	Amount	Equipment[1]	Exp. Time[2]	Bias/Comment
Cell lysis	Salt, pH, ion strength, detergents	-	Ultrasonic sonotrode	30 min	• Loss of proteoforms with a *pI* close to the pH of the extraction solution.<br>• Acidic conditions: Artificial hydrolysis of peptide bonds C-terminal to aspartate residues.<br>• Artificial modifications due to the protease inhibitor are possible (e.g., alkaline pH and high organic content).
Reduction	TCEP, 1 h, 50 °C	-	Thermomix	60 min	• Identification of more cysteine-containing proteoforms with higher residue cleavage.<br>• Potential artificial disulfides due to re-oxidation or insufficient reduction.<br>• Artificial hydrolysis of peptide bonds C-terminal to aspartate residues.<br>• Loss of proteoform information regarding disulfides.
Reduction/ Alkylation	IAA, RT, 30 min	-	Thermomix	90 min	• Identification of more cysteine-containing proteoforms with a higher sequence coverage.<br>• Artificial hydrolysis of peptide bonds C-terminal to aspartate residues.<br>• Loss of proteoform information regarding disulfides.
1D-LC	-	~10 µg	-	5 min	• Fast, simple, and cheap approach.<br>• Acceptable number of identifications with no apparent bias.<br>• Significant proteoform loss during re-solubilization in LC-MS loading buffer.
SPE	SPE material: C4, C18	500 µg	SPE cartridge	30 min	• High number of identifications and overlap coefficient with 1D-LC analysis.<br>• Fast, simple, and cheap approach.<br>• Artificial formylation events.
PEPPI	MCW AnExSP	80 µg	PAGE system	2 h	• A relatively low sample amount is required. Simple and cheap approach.<br>• Bias towards acidic proteoforms.<br>• Loss of proteoforms with alkaline *pI*, such as histones.<br>• Possibility for fractionation approaches to improve analysis depth.<br>• Possibility for targeted approaches.
GELFrEE	8% cartridge	500 µg	GELFrEE system + cartridge	2-4 h	• Artificial hydrolysis of peptide bonds C-terminal to aspartate residues.<br>• Possibility for fractionation approaches to improve the analysis depth.<br>• GELFrEE system and cartridges are no longer commercially available.

SEC	Biosep-S3000, 40% acetonitrile, 0.1% TFA	125 µg	HPLC system + column	15 min	• Expensive equipment/columns are required.<br>• Possibility for fractionation approaches to improve analysis depth
MWCO	Pore size (30/50 kDa)	500 µg	MWCO filter	3 h	• High number of identifications.<br>• Low reproducibility.
Acetonitrile depletion	Acidic (NaCl/TFA), Basic (TEAB)	500 µg	-	90 min	• High number of identifications.<br>• Bias towards proteoforms smaller than 10 kDa.<br>• Bias towards proteoforms with acidic or alkaline *pI* (acidic and basic depletion, respectively).<br>• Highly complementary to other sample preparation strategies.
2D low/low pH	PLRP-S (TFA)/ C4 (formic acid)	500 µg	HPLC system + column	2 h	• Expensive equipment/columns are required.<br>• Artificial hydrolysis of peptide bonds C-terminal to aspartate residues.<br>• High number of proteoform identifications.

[1]Only the specific equipment required for sample preparation is listed and not, for example, common lab equipment, such as a centrifuge.

[2]The experiment time refers to the duration required only for the actual sample preparation step and does not include, for example, the drying of the sample and resuspension before LC-MS/MS analysis. The time required to ensure satisfactory performance is also not included for chromatography-based methods.

Supplementary Table 8: **Overview of the sample preparation steps, critical factors regarding protein loss and potential artifacts, and possible solutions to tackle these problems**. A detailed description of the table is provided in the Supplementary Notes: Guidelines for the Sample Preparation in TDP.

Sample Preparation	Critical Factors	Observation	Possible Solution(s)
Cell lysis	Heat development during sonication	Artificial truncation of peptide bonds C-terminal to aspartate residues and, in particular, between aspartate-proline bonds	Instead of using a sonication probe for cell lysis (which can, e.g., result in the heating and mechanical stress of the sample), less intense lysis conditions, such as an ultrasonication bath, can be used
	Mild acidic conditions		Use alkaline buffers during cell lysis
	Protease inhibitor	Artificially introduced modifications (e.g., AEBSF adducts)	• Use alternative protease inhibitors<br>• Use alternative lysis solution (low concentration of organic solvent, acidic pH)<br>• Use suitable sample preparation to remove compounds of the protease inhibitor
		Compounds of the protease inhibitor may be enriched during sample preparation and observed as contamination peaks	
Sample cleanup	Precipitation	Loss of proteoforms[20,21,31]	• Optimized precipitation protocols, such as acetone precipitation with zinc sulfate[31]<br>• Avoid classical precipitation approaches, e.g., by using anion-exchange[20]<br>• Some sample preparation techniques, such as SPE or MWCO, can be used for sample purification, potentially omitting the precipitation step. This can be especially useful for low-input proteomics.
	Protein resolubilization	Protein loss during re-solubilization of the precipitated protein pellet	Incubate the protein pellet in cold (−20°C) 80% formic acid and then dilute tenfold with water.[32] Cooling of the sample is necessary to prevent artificial formylation of the proteoforms.[33]

Reduction	Elevated temperature	See above	Avoid heating during reduction, e.g., by using TCEP at room temperature[34]
	Disulfide refolding during sample preparation	Disulfides identified in database searches	Perform reduction with subsequent alkylation of the proteoforms
Alkylation	Alkylation	Side reactions of the alkylation reagent resulting in over-alkylation[37]	Adjust alkylation reagent concentration[39]
PEPPI/ GELFrEE	Elevated temperatures	See above	Avoid heating during the reduction in Laemmli buffer
	β-Mercaptoethanol/ acrylamide modifications	Covalent artificially introduced cysteine-modifications	• Use a different reduction reagent<br>• Perform reduction with subsequent alkylation of the proteoforms prior to the gel-based separation
SPE	Formic acid	Artificially introduced formylation due to high formic acid concentration at room temperature	• Work at low temperatures (4 °C)[33]<br>• Use a different ion-pairing reagents, such as TFA[35]
	Compounds in the sample	Some hydrophobic reagents, such as compounds of the protease inhibitor (e.g., pepstatin A), may be enriched during solid-phase extraction and observed as contamination peak	• Use alternative reagents<br>• Perform alternative sample preparation
2D-low/low pH-LC	Elevated Temperatures	See above	• Instead of using SpeedVac for sample concentration after first dimension fractionation, use a more gentle method, such as lyophilization[22]
	Acidic conditions		• Buffer the fractions to basic pH immediately after fractionation[22]<br>• Perform alternative 2D approaches, such as the high/low pH separation scheme[23]

Supplementary References

(1) Kaulich, P. T.; Cassidy, L.; Winkels, K.; Tholey, A. Improved Identification of Proteoforms in Top-Down Proteomics Using FAIMS with Internal CV Stepping. *Anal. Chem.* **2022**, *94* (8), 3600–3607.

(2) Fornelli, L.; Durbin, K. R.; Fellers, R. T.; Early, B. P.; Greer, J. B.; LeDuc, R. D.; Compton, P. D.; Kelleher, N. L. Advancing Top-down Analysis of the Human Proteome Using a Benchtop Quadrupole-Orbitrap Mass Spectrometer. *J. Proteome Res.* **2017**, *16* (2), 609–618.

(3) Cassidy, L.; Helbig, A. O.; Kaulich, P. T.; Weidenbach, K.; Schmitz, R. A.; Tholey, A. Multidimensional Separation Schemes Enhance the Identification and Molecular Characterization of Low Molecular Weight Proteomes and Short Open Reading Frame-Encoded Peptides in Top-down Proteomics. *J. Proteomics* **2021**, *230* (103988), 103988.

(4) LeDuc, R. D.; Fellers, R. T.; Early, B. P.; Greer, J. B.; Thomas, P. M.; Kelleher, N. L. The C-Score: A Bayesian Framework to Sharply Improve Proteoform Scoring in High-Throughput Top down Proteomics. *J. Proteome Res.* **2014**, *13* (7), 3231–3240.

(5) Melani, R. D.; Gerbasi, V. R.; Anderson, L. C.; Sikora, J. W.; Toby, T. K.; Hutton, J. E.; Butcher, D. S.; Negrão, F.; Seckler, H. S.; Srzentic, K.; Fornelli, L.; Camarillo, J. M.; LeDuc, R. D.; Cesnik, A. J.; Lundberg, E.; Greer, J. B.; Fellers, R. T.; Robey, M. T.; DeHart, C. J.; Forte, E.; Hendrickson, C. L.; Abbatiello, S. E.; Thomas, P. M.; Kokaji, A. I.; Levitsky, J.; Kelleher, N. L. The Blood Proteoform Atlas: A Reference Map of Proteoforms in Human Hematopoietic Cells. *Science (80-.).* **2022**, *375* (6579), 411–418.

(6) Guner, H.; Close, P. L.; Cai, W.; Zhang, H.; Peng, Y.; Gregorich, Z. R.; Ge, Y. MASH Suite: A User-Friendly and Versatile Software Interface for High-Resolution Mass Spectrometry Data Interpretation and Visualization. *J. Am. Soc. Mass Spectrom.* **2014**, *25* (3), 464–470.

(7) Brunner, A. M.; Lössl, P.; Liu, F.; Huguet, R.; Mullen, C.; Yamashita, M.; Zabrouskov, V.; Makarov, A.; Altelaar, A. F. M.; Heck, A. J. R. Benchmarking Multiple Fragmentation Methods on an Orbitrap Fusion for Top-down Phospho-Proteoform Characterization. *Anal. Chem.* **2015**, *87* (8), 4152–4158.

(8) Piehowski, P. D.; Petyuk, V. A.; Orton, D. J.; Xie, F.; Moore, R. J.; Ramirez-Restrepo, M.; Engel, A.; Lieberman, A. P.; Albin, R. L.; Camp, D. G.; Smith, R. D.; Myers, A. J. Sources of Technical Variability in Quantitative LC-MS Proteomics: Human Brain Tissue Sample Analysis. *J. Proteome Res.* **2013**, *12* (5), 2128–2137.

(9) Tabb, D. L.; Vega-Montoto, L.; Rudnick, P. A.; Variyath, A. M.; Ham, A. J. L.; Bunk, D. M.; Kilpatrick, L. E.; Billheimer, D. D.; Blackman, R. K.; Cardasis, H. L.; Carr, S. A.; Clauser, K. R.; Jaffe, J. D.; Kowalski, K. A.; Neubert, T. A.; Regnier, F. E.; Schilling, B.; Tegeler, T. J.; Wang, M.; Wang, P.; Whiteaker, J. R.; Zimmerman, L. J.; Fisher, S. J.; Gibson, B. W.; Kinsinger, C. R.; Mesri, M.; Rodriguez, H.; Stein, S. E.; Tempst, P.; Paulovich, A. G.; Liebler, D. C.; Spiegelman, C. Repeatability and Reproducibility in Proteomic Identifications by Liquid Chromatography-Tandem Mass Spectrometry. *J. Proteome Res.* **2010**, *9* (2), 761–776.

(10) Geis-Asteggiante, L.; Ostrand-Rosenberg, S.; Fenselau, C.; Edwards, N. J. Evaluation

of Spectral Counting for Relative Quantitation of Proteoforms in Top-down Proteomics. *Anal. Chem.* **2016**, *88* (22), 10900–10907.

(11) Ham, B. M.; Yang, F.; Jayachandran, H.; Jaitly, N.; Monroe, M. E.; Gritsenko, M. A.; Livesay, E. A.; Zhao, R.; Purvine, S. O.; Orton, D.; Adkins, J. N.; Camp, D. G.; Rossie, S.; Smith, R. D. The Influence of Sample Preparation and Replicate Analyses on HeLa Cell Phosphoproteome Coverage. *J. Proteome Res.* **2008**, *7* (6), 2215–2221.

(12) Maia, T. M.; Staes, A.; Plasman, K.; Pauwels, J.; Boucher, K.; Argentini, A.; Martens, L.; Montoye, T.; Gevaert, K.; Impens, F. Simple Peptide Quantification Approach for MS-Based Proteomics Quality Control. *ACS Omega* **2020**, *5* (12), 6754–6762.

(13) Daoudi, K.; Malosse, C.; Lafnoune, A.; Darkaoui, B.; Chakir, S.; Sabatier, J. M.; Chamot-Rooke, J.; Cadi, R.; Oukkache, N. Mass Spectrometry-Based Top-down and Bottom-up Approaches for Proteomic Analysis of the Moroccan Buthus Occitanus Scorpion Venom. *FEBS Open Bio* **2021**, *11* (7), 1867–1892.

(14) Takemori, A.; Butcher, D. S.; Harman, V. M.; Brownridge, P.; Shima, K.; Higo, D.; Ishizaki, J.; Hasegawa, H.; Suzuki, J.; Yamashita, M.; Loo, J. A.; Loo, R. R. O.; Beynon, R. J.; Anderson, L. C.; Takemori, N. PEPPI-MS: Polyacrylamide-Gel-Based Prefractionation for Analysis of Intact Proteoforms and Protein Complexes by Mass Spectrometry. *J. Proteome Res.* **2020**, *19* (9), 3779–3791.

(15) Tran, J. C.; Doucette, A. A. Multiplexed Size Separation of Intact Proteins in Solution Phase for Mass Spectrometry. *Anal. Chem.* **2009**, *81* (15), 6201–6209.

(16) Cassidy, L.; Kaulich, P. T.; Tholey, A. Depletion of High-Molecular-Mass Proteins for the Identification of Small Proteins and Short Open Reading Frame Encoded Peptides in Cellular Proteomes. *J. Proteome Res.* **2019**, *18* (4), 1725–1734.

(17) Genth, J.; Schäfer, K.; Cassidy, L.; Graspeuntner, S.; Rupp, J.; Tholey, A. Identification of Proteoforms of Short Open Reading Frame-Encoded Peptides in Blautia Producta under Different Cultivation Conditions. *Microbiol. Spectr.* **2023**, *11* (6).

(18) Wang, B.; Wang, Z.; Pan, N.; Huang, J.; Wan, C. Improved Identification of Small Open Reading Frames Encoded Peptides by Top-Down Proteomic Approaches and De Novo Sequencing. *Int. J. Mol. Sci.* **2021**, *22* (11), 5476.

(19) Takemori, A.; Kawashima, Y.; Takemori, N. Bottom-up/Cross-Linking Mass Spectrometry. *Chem. Commun.* **2022**, *58* (6), 775–778.

(20) Takemori, A.; Kaulich, P. T.; Cassidy, L.; Takemori, N.; Tholey, A. Size-Based Proteome Fractionation through Polyacrylamide Gel Electrophoresis Combined with LC-FAIMS-MS for In-Depth Top-Down Proteomics. *Anal. Chem.* **2022**, *94* (37), 12815–12821.

(21) Kline, J. T.; Belford, M. W.; Boeser, C. L.; Huguet, R.; Fellers, R. T.; Greer, J. B.; Greer, S. M.; Horn, D. M.; Durbin, K. R.; Dunyach, J. J.; Ahsan, N.; Fornelli, L. Orbitrap Mass Spectrometry and High-Field Asymmetric Waveform Ion Mobility Spectrometry (FAIMS) Enable the in-Depth Analysis of Human Serum Proteoforms. *J. Proteome Res.* **2023**, *22* (11), 3418–3426.

(22) Kaulich, P. T.; Cassidy, L.; Tholey, A. Identification of Proteoforms by Top-down Proteomics Using Two-Dimensional Low/Low PH Reversed-Phase Liquid Chromatography-Mass Spectrometry. *Proteomics* **2024**, *24* (3–4), 1–11.

(23) Wang, Z.; Ma, H.; Smith, K.; Wu, S. Two-Dimensional Separation Using High-PH and Low-PH Reversed Phase Liquid Chromatography for Top-down Proteomics. *Int. J. Mass Spectrom.* **2018**, *427* (405), 43–51.

(24) Wang, Z.; Yu, D.; Cupp-Sutton, K. A.; Liu, X.; Smith, K.; Wu, S. Development of an Online 2D Ultrahigh-Pressure Nano-LC System for High-PH and Low-PH Reversed Phase Separation in Top-Down Proteomics. *Anal. Chem.* **2020**, *92* (19), 12774–12777.

(25) Wingfield, P. T. N-Terminal Methionine Processing. *Curr. Protoc. Protein Sci.* **2017**, *2017* (April), 14–16.

(26) Bateman, A.; Martin, M. J.; Orchard, S.; Magrane, M.; Ahmad, S.; Alpi, E.; Bowler-Barnett, E. H.; Britto, R.; Bye-A-Jee, H.; Cukura, A.; Denny, P.; Dogan, T.; Ebenezer, T. G.; Fan, J.; Garmiri, P.; da Costa Gonzales, L. J.; Hatton-Ellis, E.; Hussein, A.; Ignatchenko, A.; Insana, G.; Ishtiaq, R.; Joshi, V.; Jyothi, D.; Kandasaamy, S.; Lock, A.; Luciani, A.; Lugaric, M.; Luo, J.; Lussi, Y.; MacDougall, A.; Madeira, F.; Mahmoudy, M.; Mishra, A.; Moulang, K.; Nightingale, A.; Pundir, S.; Qi, G.; Raj, S.; Raposo, P.; Rice, D. L.; Saidi, R.; Santos, R.; Speretta, E.; Stephenson, J.; Totoo, P.; Turner, E.; Tyagi, N.; Vasudev, P.; Warner, K.; Watkins, X.; Zaru, R.; Zellner, H.; Bridge, A. J.; Aimo, L.; Argoud-Puy, G.; Auchincloss, A. H.; Axelsen, K. B.; Bansal, P.; Baratin, D.; Batista Neto, T. M.; Blatter, M. C.; Bolleman, J. T.; Boutet, E.; Breuza, L.; Gil, B. C.; Casals-Casas, C.; Echioukh, K. C.; Coudert, E.; Cuche, B.; de Castro, E.; Estreicher, A.; Famiglietti, M. L.; Feuermann, M.; Gasteiger, E.; Gaudet, P.; Gehant, S.; Gerritsen, V.; Gos, A.; Gruaz, N.; Hulo, C.; Hyka-Nouspikel, N.; Jungo, F.; Kerhornou, A.; Le Mercier, P.; Lieberherr, D.; Masson, P.; Morgat, A.; Muthukrishnan, V.; Paesano, S.; Pedruzzi, I.; Pilbout, S.; Pourcel, L.; Poux, S.; Pozzato, M.; Pruess, M.; Redaschi, N.; Rivoire, C.; Sigrist, C. J. A.; Sonesson, K.; Sundaram, S.; Wu, C. H.; Arighi, C. N.; Arminski, L.; Chen, C.; Chen, Y.; Huang, H.; Laiho, K.; McGarvey, P.; Natale, D. A.; Ross, K.; Vinayaka, C. R.; Wang, Q.; Wang, Y.; Zhang, J. UniProt: The Universal Protein Knowledgebase in 2023. *Nucleic Acids Res.* **2023**, *51* (D1), D523–D531.

(27) Fuchs, S.; Kucklick, M.; Lehmann, E.; Beckmann, A.; Wilkens, M.; Kolte, B.; Mustafayeva, A.; Ludwig, T.; Diwo, M.; Wissing, J.; Jänsch, L.; Ahrens, C. H.; Ignatova, Z.; Engelmann, S. Towards the Characterization of the Hidden World of Small Proteins in Staphylococcus Aureus, a Proteogenomics Approach. *PLoS Genet.* **2021**, *17* (6), 1–26.

(28) Cassidy, L.; Kaulich, P. T.; Tholey, A. Proteoforms Expand the World of Microproteins and Short Open Reading Frame-Encoded Peptides. *iScience* **2023**, *26* (2), 106069.

(29) Stathopulos, P. B.; Scholz, G. A.; Hwang, Y.; Rumfeldt, J. A. O.; Lepock, J. R.; Meiering, E. M. Sonication of Proteins Causes Formation of Aggregates That Resemble Amyloid. *Protein Sci.* **2004**, *13* (11), 3017–3027.

(30) Yang, Z.; Shen, X.; Chen, D.; Sun, L. Toward a Universal Sample Preparation Method for Denaturing Top-Down Proteomics of Complex Proteomes. *J. Proteome Res.* **2020**, *19* (8), 3315–3325.

(31) Baghalabadi, V.; Doucette, A. A. Mass Spectrometry Profiling of Low Molecular Weight Proteins and Peptides Isolated by Acetone Precipitation. *Anal. Chim. Acta* **2020**, *1138*, 38–48.

(32) Doucette, A. A.; Vieira, D. B.; Orton, D. J.; Wall, M. J. Resolubilization of Precipitated Intact Membrane Proteins with Cold Formic Acid for Analysis by Mass Spectrometry.

J. Proteome Res. **2014**, *13* (12), 6001–6012.

(33) Zheng, S.; Doucette, A. A. Preventing N- and O-Formylation of Proteins When Incubated in Concentrated Formic Acid. *Proteomics* **2016**, *16* (7), 1059–1068.

(34) Guo, Y.; Yu, D.; Cupp-Sutton, K. A.; Liu, X.; Wu, S. Optimization of Protein-Level Tandem Mass Tag (TMT) Labeling Conditions in Complex Samples with Top-down Proteomics. *Anal. Chim. Acta* **2022**, *1221* (May).

(35) Maráková, K.; Renner, B. J.; Thomas, S. L.; Opetová, M.; Tomašovský, R.; Rai, A. J.; Schug, K. A. Solid Phase Extraction as Sample Pretreatment Method for Top-down Quantitative Analysis of Low Molecular Weight Proteins from Biological Samples Using Liquid Chromatography – Triple Quadrupole Mass Spectrometry. *Anal. Chim. Acta* **2023**, *1243*.

(36) Huang, C. F.; Kline, J. T.; Negrão, F.; Robey, M. T.; Toby, T. K.; Durbin, K. R.; Fellers, R. T.; Friedewald, J. J.; Levitsky, J.; Abecassis, M. M. I.; Melani, R. D.; Kelleher, N. L.; Fornelli, L. Targeted Quantification of Proteoforms in Complex Samples by Proteoform Reaction Monitoring. *Anal. Chem.* **2024**, *96* (8), 3578–3586.

(37) Kaulich, P. T.; Winkels, K.; Kaulich, T. B.; Treitz, C.; Cassidy, L.; Tholey, A. MSTopDiff: A Tool for the Visualization of Mass Shifts in Deconvoluted Top-Down Proteomics Data for the Database-Independent Detection of Protein Modifications. *J. Proteome Res.* **2022**, *21* (1), 20–29.

(38) Lex, A.; Gehlenborg, N.; Strobelt, H.; Vuillemot, R.; Pfister, H. UpSet: Visualization of Intersecting Sets. *IEEE Trans. Vis. Comput. Graph.* **2014**, *20* (12), 1983–1992.

(39) Suttapitugsakul, S.; Xiao, H.; Smeekens, J.; Wu, R. Evaluation and Optimization of Reduction and Alkylation Methods to Maximize Peptide Identification with MS-Based Proteomics. *Mol. Biosyst.* **2017**, *13* (12), 2574–2582.
