# Supplementary material for: Influence of different sample preparation approaches on proteoform identification by top-down proteomics
Source: Nat Methods. 2024 Oct 22;21(12):2397–407. doi: 10.1038/s41592-024-02481-6 (PMC11621018; doi:10.1038/s41592-024-02481-6)
Supplement: Supplementary file 2 — Reporting Summary [file 41592_2024_2481_MOESM2_ESM.pdf]

nature portfolio

Corresponding author(s): Andreas Tholey

Last updated by author(s): Feb 27, 2024

Reporting Summary

Nature Portfolio wishes to improve the reproducibility of the work that we publish. This form provides structure for consistency and transparency in reporting. For further information on Nature Portfolio policies, see our Editorial Policies and the Editorial Policy Checklist.

Statistics

For all statistical analyses, confirm that the following items are present in the figure legend, table legend, main text, or Methods section.

n/a	Confirmed	
☐	☒	The exact sample size (*n*) for each experimental group/condition, given as a discrete number and unit of measurement
☐	☒	A statement on whether measurements were taken from distinct samples or whether the same sample was measured repeatedly
☒	☐	The statistical test(s) used AND whether they are one- or two-sided<br>*Only common tests should be described solely by name; describe more complex techniques in the Methods section.*
☒	☐	A description of all covariates tested
☒	☐	A description of any assumptions or corrections, such as tests of normality and adjustment for multiple comparisons
☒	☐	A full description of the statistical parameters including central tendency (e.g. means) or other basic estimates (e.g. regression coefficient) AND variation (e.g. standard deviation) or associated estimates of uncertainty (e.g. confidence intervals)
☒	☐	For null hypothesis testing, the test statistic (e.g. *F*, *t*, *r*) with confidence intervals, effect sizes, degrees of freedom and *P* value noted<br>*Give P values as exact values whenever suitable.*
☒	☐	For Bayesian analysis, information on the choice of priors and Markov chain Monte Carlo settings
☒	☐	For hierarchical and complex designs, identification of the appropriate level for tests and full reporting of outcomes
☒	☐	Estimates of effect sizes (e.g. Cohen's *d*, Pearson's *r*), indicating how they were calculated

Our web collection on statistics for biologists contains articles on many of the points above.

Software and code

Policy information about availability of computer code

Data collection	Thermo Scientific™ Orbitrap™ Tribrid™ Series 3.4 instrument control application (v3.4.3072.18)
Data analysis	(i) ProSightPD (v4.2) within the Proteome Discoverer Suite (v3.0.0.757) (commercial, Thermo Scientific); (ii)FLASHDeconv (v2.0, default settings),Jeong et al., Cell Syst. 2020, 10 (2), 213-218.e6; (iii) MSTopDIFF (v1.1.0, default settings); Kaulich et al., J Proteome Res 2022, 21: 20-29; (iv) Python V11.3.2, including Pyteomics V4.6, and PyOpenMS V.3.1.0

For manuscripts utilizing custom algorithms or software that are central to the research but not yet described in published literature, software must be made available to editors and reviewers. We strongly encourage code deposition in a community repository (e.g. GitHub). See the Nature Portfolio guidelines for submitting code & software for further information.

Data

Policy information about availability of data

All manuscripts must include a data availability statement. This statement should provide the following information, where applicable:

- Accession codes, unique identifiers, or web links for publicly available datasets
- A description of any restrictions on data availability
- For clinical datasets or third party data, please ensure that the statement adheres to our policy

ProteomeXchange Consortium via the PRIDE partner repository with the dataset identifier PXD049969. Human protein database including all known modifications for database search can be downloaded as XML file from UniProt (https://www.uniprot.org, taxon ID 9606).

Human research participants

Policy information about studies involving human research participants and Sex and Gender in Research.

Reporting on sex and gender	n/a
Population characteristics	n/a
Recruitment	n/a
Ethics oversight	n/a

Note that full information on the approval of the study protocol must also be provided in the manuscript.

Field-specific reporting

Please select the one below that is the best fit for your research. If you are not sure, read the appropriate sections before making your selection.

☒ Life sciences ☐ Behavioural & social sciences ☐ Ecological, evolutionary & environmental sciences

For a reference copy of the document with all sections, see nature.com/documents/nr-reporting-summary-flat.pdf

Life sciences study design

All studies must disclose on these points even when the disclosure is negative.

Sample size	Cell culture based experiments: one batch (biological replicate) of Caco-2 cells was used for all experiments performed in this study. For the optimization of the LC-MS workflow, the influence of the number of replicate measurements on the identifications were investigated. Three replicates were a compromise to cover a high number of proteoforms and to investigate the reproducibility while being economical with measurement time.<br><br>For investigating the influence of sample preparation (i.e., cell lysis, enrichment of suitable proteoforms, proteoform fractionation), the respective sample preparation was independently performed three times.
Data exclusions	only protein/proteoform identifications matching the quality criteria/tresholds given in the manuscript are reported (1% false discovery rate; C-score >40)
Replication	Three technical replicates were performed as standard, unless otherwise stated. All technical replicates performed were included in this study, unless a LC-MS run was interrupted due to technical issues (i.e., instrument performance); in the latter case, the entire measurement series was discarded and repeated.
Randomization	No randomization was necessary as this was a qualitative study, based on the performance of technical replicates out of singe biological replicate (single batch cell culture).
Blinding	No blinding was necessary as this was a qualitative study, based on the performance of technical replicates out of singe biological replicate (single batch cell culture).

Reporting for specific materials, systems and methods

We require information from authors about some types of materials, experimental systems and methods used in many studies. Here, indicate whether each material, system or method listed is relevant to your study. If you are not sure if a list item applies to your research, read the appropriate section before selecting a response.

Materials & experimental systems

n/a	Involved in the study	
☒	☐	Antibodies
☐	☒	Eukaryotic cell lines
☒	☐	Palaeontology and archaeology
☒	☐	Animals and other organisms
☒	☐	Clinical data
☒	☐	Dual use research of concern

Methods

n/a	Involved in the study	
☒	☐	ChIP-seq
☒	☐	Flow cytometry
☒	☐	MRI-based neuroimaging

Eukaryotic cell lines

Policy information about cell lines and Sex and Gender in Research

Cell line source(s)	Human Caco 2 (colon adenocarcinoma) cells / European Collection of Cell Cultures
Authentication	the cell line was not authenticicated
Mycoplasma contamination	not tested
Commonly misidentified lines (See ICLAC register)	The cell line used is not listed in the ICLAC register (release 2024, April, 26).
